# *DCST1-AS1* Promotes TGF-β-Induced Epithelial–Mesenchymal Transition and Enhances Chemoresistance in Triple-Negative Breast Cancer Cells via ANXA1

**DOI:** 10.3389/fonc.2020.00280

**Published:** 2020-03-12

**Authors:** Li Tang, Yuli Chen, Huanhuan Chen, Pan Jiang, Linping Yan, Dongping Mo, Xun Tang, Feng Yan

**Affiliations:** ^1^Department of Clinical Laboratory, Jiangsu Cancer Hospital & Jiangsu Institute of Cancer Research & the Affiliated Cancer Hospital of Nanjing Medical University, Nanjing, China; ^2^Department of Clinical Laboratory, Nanjing Qixia District Hospital, Nanjing, China; ^3^The Fourth Clinical Medical School, Nanjing Medical University, Nanjing, China

**Keywords:** long non-coding RNA, ANXA1, EMT, migration, invasion, breast cancer

## Abstract

Triple-negative breast cancer (TNBC) is a highly metastatic breast cancer subtype, and the primary systemic treatment strategy involves conventional chemotherapy. DC-STAMP domain containing 1-antisense 1 (*DCST1-AS1*) is a long non-coding RNA that promotes TNBC migration and invasion. Studying the role of *DCST1-AS1* in promoting epithelial–mesenchymal transition (EMT) and chemoresistance will provide a new strategy for TNBC therapy. In the present study, we found that *DCST1-AS1* regulates the expression or secretion of EMT-related proteins E-cadherin, snail family zinc finger 1 (SNAI1), vimentin, matrix metallopeptidase 2 (MMP2), and matrix metallopeptidase 9 (MMP9). Interference with *DCST1-AS1* impaired TGF-β-induced TNBC cell invasion and migration. *DCST1-AS1* directly binds to ANXA1 in BT-549 cells and affects the expression of ANXA1. *DCST1-AS1* enhances TGF-β/Smad signaling in BT-549 cells through ANXA1 to promote EMT. The combination of *DCST1-AS1* and ANXA1 also contributes to enhancement of the resistance of BT-549 cells to doxorubicin and paclitaxel. In conclusion, *DCST1-AS1* promotes TGF-β-induced EMT and enhances chemoresistance in TNBC cells through ANXA1, and therefore represents a potentially promising target for metastatic breast cancer therapy.

## Introduction

Breast cancer is a highly heterogeneous disease. The classification of breast cancer is generally based on histological subtypes associated with unique clinical manifestations or molecular features identified by gene expression analysis. The most popular classification is the one based on molecular subtypes determined by gene expression profiling proposed by the expert panel at the St Gallen International Breast Cancer Conference in 2013 (1) and includes luminal A, luminal B, human epidermal growth factor receptor 2-positive (HER2+) overexpressing, and basal-like (BL) subtypes. Luminal tumors express the estrogen and progesterone receptors. Patients with luminal tumors generally carry a good prognosis and respond well to hormone therapy (2). Patients with HER2-overexpressing tumors (estrogen receptor negative, progesterone receptor negative, HER2+) have a poor prognosis but are sensitive to therapies targeting HER2 receptors, such as trastuzumab or the dual EGFR/HER2 kinase inhibitor, lapatinib (3). Compared to the other tumor subtypes, BL breast cancer patients have a more aggressive clinical course, higher risk of recurrence, and lower survival rate. Approximately 77% of BL tumors exhibit loss of expression of the estrogen receptor, progesterone receptor, and HER2, and are referred to as triple-negative breast cancer (TNBC) (4). The TNBC subtype is characterized by enhanced invasiveness and formation of distant metastases whose migration characteristics are associated with the acquisition of mesenchymal phenotype by epithelial cells (5). Unfortunately, due to the lack of expression of hormone receptors and the absence of HER2 protein overexpression, there is currently no targeted therapy for this breast cancer subtype, and chemotherapy remains the standard treatment for patients with triple-negative disease.

Annexin A1 (ANXA1) is a member of the Ca^2+^-dependent phospholipid-binding protein family and has long been classified as an anti-inflammatory protein due to its control over leukocyte-mediated immune responses (6). Further studies have shown that in addition to inflammatory responses, ANXA1 also participates in key intracellular signaling pathways through protein cascades and plays a regulatory role in cancer cell proliferation, adhesion, apoptosis, cytoskeletal protein reorganization, angiogenesis, and invasion and metastasis (7). Changes in ANXA1 expression are associated with specific types of tumors, and the expression of ANXA1 has been studied in a wide range of cancers: ANXA1 is upregulated in lung cancer, colorectal cancer, hepatocellular carcinoma, pancreatic cancer, multiple myeloma, and melanoma, while in other types of cancers such as esophageal squamous cell carcinoma, gastric cancer, head, and neck squamous cell carcinoma, and prostate cancer, ANXA1 is downregulated or absent (8). It is noteworthy that ANXA1 shows differences in expression levels in the highly heterogeneous breast cancer: Patients with luminal breast cancer showed loss of ANXA1 expression, whereas patients with BRCA1/2 mutation, triple-negative, and poorly differentiated breast cancer showed ANXA1 upregulation (9). Clinical macro data confirmed that elevated ANXA1 expression was significantly associated with higher pathological grades and worse breast cancer-associated survival (10).

Our previous research confirmed that the long non-coding RNA, DC-STAMP domain containing 1-antisense 1 (*DCST1-AS1*), is highly expressed in TNBC tissues and is associated with poor histopathological grade and distant metastasis. We also determined that *DCST1-AS1* regulates insulin-like growth factor-2 mRNA-binding protein 1 (IGF2BP1) through *miR-873-5p* to promote TNBC cell proliferation and metastasis (11). In this study, we found that *DCST1-AS1* binds to ANXA1 to promote TGF-β-induced epithelial–mesenchymal transition (EMT) processes in breast cancer cells. *DCST1-AS1* also enhanced the chemoresistance of BT-549 cells to doxorubicin and paclitaxel through ANXA1.

## Materials and Methods

### Materials

Human transforming growth factor-β1 (TGF-β1) was obtained from R&D Systems (Minneapolis, MN, USA). Corning BioCoat^TM^ Tumor Invasion 24-well plate was purchased from Corning Incorporated (Corning, NY, USA). Leibovitz's L-15 medium, trypsin-EDTA (0.25%), and fetal bovine serum (FBS) were procured from GIBCO BRL (Grand Island, NY, USA). cOmplete^TM^, EDTA-free Protease Inhibitor Cocktail, PhosSTOP^TM^ phosphatase inhibitor Cocktail, and TRIzol reagent were purchased from Sigma-Aldrich (St. Louis, MO, USA). RIPA lysate, QuicBlock^TM^ Blocking Buffer for Immunostaining, Immunostaining Permeabilization Solution with Saponin, Immunostaining Permeabilization Solution with Triton X-100, and Immunofluorescence Staining Kit with Cy3-Labeled Goat Anti-Rabbit IgG were purchased from Beyotime (Shanghai, China). Primary rabbit antibodies anti-ANXA1 and anti-SNAI1 were purchased from Abcam (Cambridge, MA, USA). Primary rabbit antibodies including anti-vimentin, E-cadherin, matrix metallopeptidase 2 (MMP2), matrix metallopeptidase 9 (MMP9), Smad2, and phospho-Smad2 Ser465/467 (p-Smad2) were purchased from Cell Signaling Technology (Danvers, MA, USA). *DCST1-AS1* stable knocked down cell lines were generated using lentivirus-mediated transduction using synthetic short hairpin RNA (shRNA) oligonucleotides (GeneChem, Shanghai, China) according to the manufacturer's protocols. Stable *DCST1-AS1*-overexpressing cell lines were produced by lentivirus-mediated transduction (Genepharma, Shanghai, China) according to the manufacturer's protocols. Puromycin (5 μg/ml) used to screen stable lentivirus-transfected cell lines was purchased from Solarbio (Beijing, China). Small interfering RNA (siRNA) oligonucleotides targeting *ANXA1* and non-targeting siRNA were purchased from GenePharma (Shanghai, China). The full-length sequence of *DCST1-AS1* was amplified *in vitro* and subcloned into the *Eco*RI and *Hin*dIII sites of the pUC57 vector, and the resulting vector was named pUC57-DCST1-AS1 (Supplementary Material 1). The cDNA sequence of *ANXA1* was amplified *in vitro* and subcloned into the *Bam*HI and *Eco*RI sites of the pcDNA3.1 vector, and the resulting vector was named pcDNA3.1-ANXA1 (Supplementary Material 2). Transfections with DNA were performed using Lipofectamine 3000 reagent (Invitrogen, Carlsbad, CA, USA). Transfections with siRNA were performed using Lipofectamine RNAiMAX Transfection Reagent (Invitrogen). Paclitaxel was purchased from Peking Union Pharmaceutical Factory (Beijing, China). Doxorubicin hydrochloride for injection was purchased from Main Luck Pharmaceuticals Inc. (Shenzhen, China). Cell Counting Kit-8 (CCK-8) was purchased from Dojindo Laboratories (Kyushu Island, Japan).

### Cell Culture

Breast cancer cell lines MDA-MB-231, BT-549, T-47D, and MCF7 were obtained from the Chinese Academy of Sciences Cell Bank (Shanghai, China). MDA-MB-231 cells were cultured as monolayers in Leibovitz's L-15 medium supplemented with L-glutamine, sodium pyruvate, and 10% FBS at 37°C in a humidified atmosphere without CO_2_ and allowing exchange with air. BT-549, T-47D, and MCF7 cells were cultured in RPMI 1640 medium containing 10% FBS at 37°C in humidified atmosphere containing 5% CO_2_. In our previous study (11), we had generated the following stably transfected cell lines: BT-549-sh and MDA-231-sh are stably transfected cell lines derived from lentiviral infection of BT-549 and MDA-MB-231 cells with *DCST1-AS1* interference fragment. BT-549-NC and MDA-231-NC are stably transfected cell lines derived from BT-549 and MDA-MB-231 cells following infection with lentivirus carrying unrelated fragments and served as negative controls. MDA-231-exp is a stably transfected cell line obtained by infecting MDA-MB-231 cells with lentivirus carrying the *DCST1-AS1* overexpression vector. MDA-231-eNC is a stably transfected cell line obtained by infecting MDA-MB-231 with lentivirus carrying an empty vector and served as a negative control. In this study, we used the same method to obtain a stably transfected cell line MCF7-exp overexpressing *DCST1-AS1* and its negative control MCF7-NC. Methods for the preparation of the stably transfected cell lines are provided in Supplementary Material 3. All cell lines were subjected to morphological examination, growth curve determination, and mycoplasma detection prior to the study.

### RNA Preparation and Quantitative Real-time PCR

Total RNAs were isolated from BT-549, MDA-MB-231, T-47D, and MCF7 using TRIzol according to the manufacturer's instructions. The cDNAs used for real-time PCR were obtained from the purified RNA using a PrimeScript RT Reagent Kit (TaKaRa, Tokyo, Japan). A two-step PCR was used for PCR amplification at a *T*_m_ of 57°C using the following primers purchased from Sangon (Shanghai, China): *DCST1-AS1* Forward: 5′-CCACTCACCAGCTTCTTC-3′; Reverse: 5′-CTTCTGCTATGTCTCACCC-3′. ANXA1 Forward: 5′-TGATGAACTTCGTGCTG-3′; Reverse: 5′-TGGTTTGCTTGTGGC-3′. The 18S rRNA was used to calculate the relative gene expression.

### Immunofluorescence Staining

Sterile slides were placed in a 24-well plate, and the cells were plated to coat the slides. When the cell reached about 60% confluence, serum-free medium was added and the cells were serum starved for 24 h. Finally, TGF-β (5 ng/ml) was added and the cells were induced for 24 h. The cells were then washed thrice with PBS and fixed with 4% paraformaldehyde for 20 min. Then, the cells were washed with PBS again and stabilized in Saponin (E-cadherin) or 0.5% Triton X-100 (vimentin) for 20 min. After washing thrice with PBS, the cells were immunostained by treating with QuicBlock^TM^ Blocking Buffer for 15 min and then incubated with anti-E-cadherin (1:200) or anti-vimentin (1:100) antibodies overnight at 4°C. After washing, the cells were incubated with Cy3-labeled anti-rabbit IgG antibody for 60 min in the dark and counterstained with Hoechst 33,342. The cells were observed and photographed with a confocal fluorescence microscope (LSM880, Zeiss, Jena, Germany).

### Cell Migration Assay

The effect of *DCST1-AS1* on the TGF-β-induced migration of TNBC cells was analyzed using wound healing assays. Cells were resuspended in serum-free medium containing TGF-β (5 ng/ml) and plated in 6-well plates at a density of 2.5 × 10^5^ cells per well. After incubating the cells for 24 h, artificial wounds were made on cell monolayer using a sterile 200-μl pipette tip. The cells were washed thoroughly with PBS to remove the cells in suspension, and fresh medium was added to continue the culture. Snapshot images were captured at 0 and 12 h using a Leica DMi8 inverted microscope (Wetzlar, Germany) to quantify the rate of wound closure and cell migration. To determine the statistical significance of the data, we analyzed the single-layer wound healing assay by processing the image using a computational tool (Image J software).

### Cell Invasion Assay

Cell invasion assays were carried out using modified Boyden chambers consisting of transwell membrane filter (8 μm pore size) inserts in 24-well tissue culture plates. Following induction with TGF-β (5 ng/ml) for 24 h, the cells were resuspended at a density of 2.5 × 10^5^ cells/ml. A total of 200 μl of the cell suspension was added to the upper chamber, and the FBS concentration in the culture medium was adjusted to 1%. A total of 500 μl of 10% FBS-containing medium was added to the lower chamber. After incubating for 18 h, the non-invasive cells and matrigel matrix were removed from the inside of the insert using a cotton swab. The invading cells were subjected to crystal violet staining and photographed using an inverted microscope.

### Enzyme-Linked Immunosorbent Assay (ELISA)

The cells were resuspended in serum-free medium and plated in 96-well plates at a cell density of 20,000 cells per well and incubated overnight. Following treatment with TGF-β (5 ng/ml) for 24 h, the cell culture supernatants were collected, and the expression of MMP2 and MMP9 was analyzed by ELISA using Human MMP9 and Human MMP2 ELISA kits (RayBiotech, Norcross, GA, USA) according to the manufacturer's instructions. These assays use human MMP2 or MMP9-specific antibody-coated 96-well plates. The standards and samples were added to the wells, and the MMP2 or MMP9 present in the sample bound to the antibodies immobilized in the well. The wells were washed and biotinylated anti-human MMP2 or MMP9 antibodies were added. After washing off the unbound biotinylated antibodies, HRP-conjugated streptavidin was added to the well. The wells were washed again, and a TMB substrate solution was added to the wells. The color development is proportional to the amount of bound MMP2 or MMP9. The stop solution changed the color from blue to yellow and intensity of the color was measured at 450 nm.

### RNA Pulldown and Mass Spectrometry

The RNA pulldown assay was performed as described below. The sense and antisense strands of *DCST1-AS1* were obtained by *in vitro* transcription, and a single dethiobiotinylated cytidine diphosphate was linked to the 3′ end of the RNA strand using T4 RNA ligase. The biotinylated RNAs were then incubated with BT-549 protein lysates to form RNA–protein complexes and combined with streptavidin-labeled magnetic beads to separate the complexes from other components in the incubation solution. After elution, the proteins pulled down by the sense or antisense *DCST1-AS1* were analyzed by sodium dodecyl sulfate polyacrylamide gel electrophoresis (SDS-PAGE). The pulled-down protein solution was subjected to enzymatic treatment and analyzed with a Triple TOF^TM^ 5600-plus instrument (AB Sciex, Redwood City, CA, USA) and the results were analyzed using Proteinpilot software. *DCST1-AS1* was amplified with the following primer sequence: Forward: 5′-taatacgactcactatagggaaagcccgggagcgcgcagacttggctgtgcg-3′; Reverse: 5′-TTTTTTCACACTTTACAGAGTTTGTTTAATGT-3′. Antisense *DCST1-AS1* was amplified with the following primer sequences: Forward: 5′-taatacgactcactatagggTTTTTTCACACTTTACAGAGTTTGTTTAATGT-3′; Reverse: 5′-aaagcccgggagcgcgcagacttggctgtgcg-3′.

### RNA Immunoprecipitation Assay

RNA immunoprecipitation experiments were performed using the Magna RIP RNA-Binding Protein Immunoprecipitation kit (Merck Millipore, Billerica, MA, USA). Briefly, BT-549 cells were harvested and RIP cell lysis buffer was added to the cells. The stock solution of magnetic beads was washed thoroughly and resuspended in the washing buffer. Anti-ANXA1 or IgG (as control) antibodies were added to bind to the magnetic beads. The supernatant from the BT-549 cell lysate was added to the magnetic bead–antibody complex and incubated at 4°C for 3 h. A magnetic frame was used to isolate the RNA bound to the magnetic bead–antibody complex. RNA was eluted from the magnetic beads and purified. RT-PCR was used to detect *DCST1-AS1* precipitated by the antibodies.

### Western Blot Analysis

Protease and phosphatase inhibitor cocktail tablets were added to RIPA buffer according to the manufacturer's instructions. Cells were washed with PBS and lysed, and the protein concentration in the lysates was measured using a standard Bradford Assay Kit (KeyGEN, Nanjing, China). Total proteins were separated on 8 or 10% SDS-PAGE, and transferred to PVDF membranes (Millipore, Tullagreen, Republic of Ireland) using a Trans-Blot SD Semi-Dry Transfer Cell (Bio-Rad, Hercules, CA, USA). After blocking with 5% BSA in TBST, the membranes were separately incubated overnight at 4°C with antibodies against ANXA1, vimentin, E-cadherin, SNAI1, Smad2, p-Smad2, MMP2, and MMP9. The membranes were washed and incubated with horseradish peroxidase-conjugated secondary antibodies for 1 h. The bands were detected using a SuperSignal West Femto Maximum Sensitivity Substrate Kit (Thermo Scientific, Rockford, IL, USA), the images were acquired using a SYNGENE G: BOX chemiXR5 system (Cambridge, UK), and the results were analyzed using Gel-Pro32 software. GAPDH expression was used as an internal control to standardize the relative expression levels of the proteins.

### Cell Viability Analysis

CCK-8 was used to determine the drug resistance of BT-549 cells. Cells were counted following trypan blue staining and plated in 96-well plates at 5,000 cells per well. Following 24 h of incubation, serial dilutions of doxorubicin (dissolved in DMSO) and paclitaxel (dissolved in absolute ethanol) were added to the cells in complete medium for different concentrations. An equal volume of corresponding vehicle was added to the wells labeled with drug concentration 0 as controls. Further, wells with the reagent combinations but without any cells served as blanks. After 48 h of treatment, the culture solution in each well was replaced with 100 μl of 10% reaction solution. After incubation for 3 h, the OD value of the viable cells was measured at 450 nm, and the cell viability was calculated according to the following formula:

$$\begin{array}{l} \text{Cell viability }(\%)\\ =\frac{\text{Drug test well }-\text{ Average of blank wells}}{\text{Average of control wells }-\text{ Average of blank wells}} \end{array}$$

### Statistical Analysis

All data presented are representative of at least three independent experiments. Student's *t*-test was used to analyze the results of wound healing experiments, cell invasion experiments, Western blotting, ELISA, and RT-PCR. Two-way analysis of variance (ANOVA) was performed to compare the data between groups in the cell viability analyses. *p* < 0.05 were considered statistically significant (*p < 0.05, **p < 0.01, ***p < 0.001). Dose–response curves were plotted using GraphPad Prism 8 software (GraphPad Software, San Diego, CA, USA), and IC_50_ values were calculated using non-linear regression, and other statistical analyses were performed using SPSS 22.0 (SPSS, Chicago, Illinois, USA).

## Results

### *DCST1-AS1* Affects TGF-β-Induced Vimentin and E-Cadherin Expression in TNBC Cells

**Figure 1:**
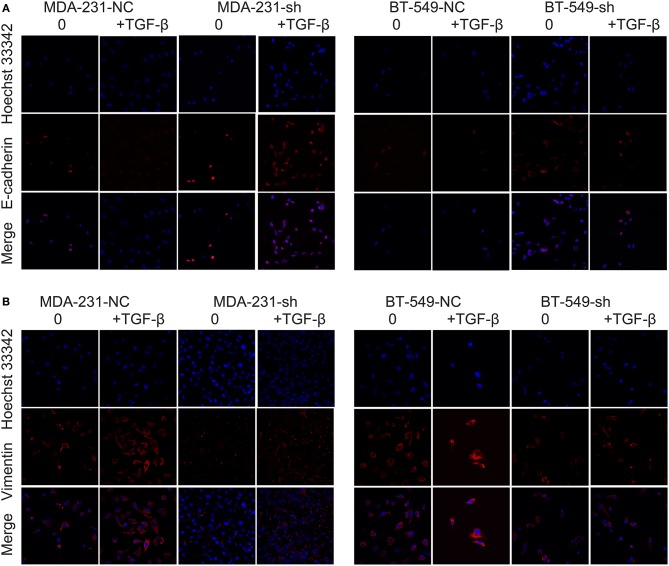
Confocal microscopy images of immunofluorescence staining showing the effect of *DCST1-AS1* on TGF-β-induced epithelial–mesenchymal transition. **(A)** The expression of E-cadherin following TGF-β stimulation for 24 h was higher in BT-549-sh and MDA-231-sh cells compared to the negative control cells. NC, negative control. **(B)** The expression of vimentin in BT-549-sh and MDA-231-sh cells following TGF-β stimulation for 24 h was lower in BT-549-sh and MDA-231-sh cells compared to the negative control cells.

Our previous studies confirmed that *DCST1-AS1* is highly expressed in BT-549 and MDA-MD-231 cells, especially in BT-549 cells. According to the information provided by ATCC for the cells, BT-549 and MDA-MB-231 are epithelial-derived breast cancer cell lines and have the morphology of epithelial cells. TGF-β has been shown to induce EMT in BT-549 and MDA-MB-231 cells *in vitro* (12). We obtained *DCST1-AS1* stable knockdown cell lines and negative controls: BT-549-sh and BT-549-NC; MDA-231-sh and MDA-231-NC. RT-PCR was used to confirm the knockdown efficiency of *DCST1-AS1* (Supplementary Figure 1). Subsequently, we treated BT-549-sh, BT-549-NC, MDA-231-sh, and MDA-231-NC with or without 5 ng/ml TGF-β for 24 h, and analyzed the changes in expression of vimentin and E-cadherin using immunofluorescence staining. We observed higher levels of E-cadherin expression in the cells with *DCST1-AS1* knockdown (BT-549-sh and MDA-231-sh) compared to the negative control cells, while vimentin expression was lower in these cells (Figures 1A,B). These changes are more pronounced in cells induced by TGF-β. These results indicate that *DCST1-AS1* affects TGF-β-induced vimentin and E-cadherin expression in BT-549 and MDA-MB-231 cells.

### *DCST1-AS1* Promotes TGF-β-Induced Migration and Invasion in TNBC Cells

**Figure 2:**
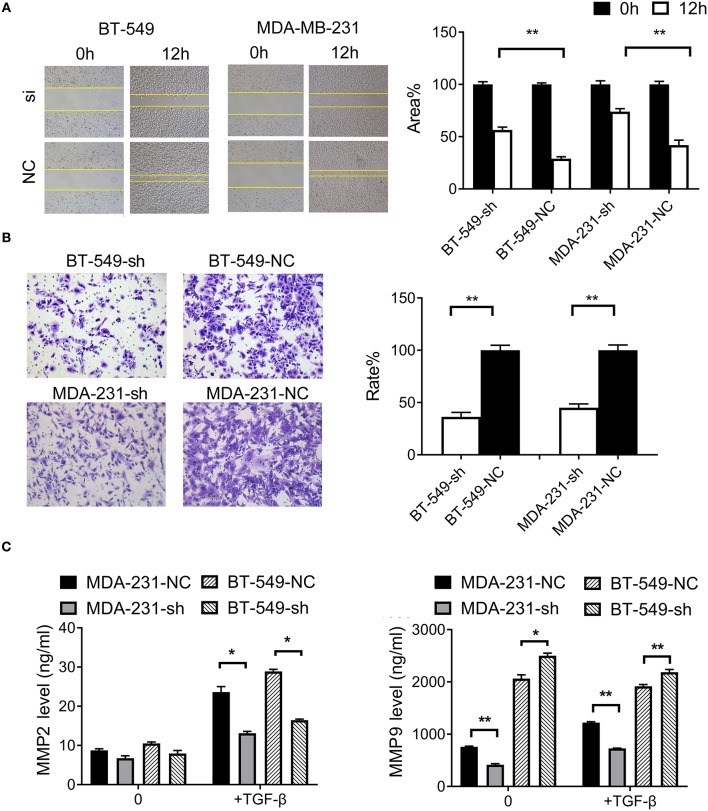
Effects of *DCST1-AS1* on TGF-β-induced migration and invasion of triple-negative breast cancer cells. **(A)** Wound healing assays were used to measure cell migration ability after 24 h of stimulation with TGF-β (5 ng/ml). The migration rate of BT-549-sh and MDA-231-sh cells was slower than that of the negative control cells, BT-549-NC, and MDA-231-NC. Images were obtained at 0 and 12 h using an inverted microscope. **(B)** Transwell chambers were used to analyze cell invasiveness after 24 h of stimulation with TGF-β (5 ng/ml). Compared to the negative control cells, fewer cells invaded the matrigel matrix in the case of BT-549-sh and MDA-231-sh. Images were obtained after 18 h using an inverted microscope. **(C)** Enzyme-linked immunosorbent assay was used to detect level of MMP2 and MMP9 in cell culture supernatants after 24 h of stimulation with TGF-β (5 ng/ml). *p < 0.05, **p < 0.01.

To examine the effect of *DCST1-AS1* on TGF-β-induced cell migration, we performed wound healing experiments with BT-549-sh, BT-549-NC, MDA-231-sh, and MDA-231-NC cells grown as confluent monolayers. After induction of the cells with TGF-β, images were captured at 0 and 12 h using an inverted microscope (Figure 2A). Compared with the negative control cells, the mobility of BT-549 and MDA-MB-231 cells was significantly slower following knockdown of *DCST1-AS1*. The open wound areas in BT-549-sh and MDA-231-sh were about 56.5% and 73.9%, respectively, which was significantly higher than that in BT-549-NC (about 29.0%) and MDA-231-NC (about 41.9%). These data showed that *DCST1-AS1* promotes TGF-β-induced migration of BT-549 and MDA-MD-231.

To investigate the effect of *DCST1-AS1* on TGF-β-induced cell invasion, we performed transwell experiments with BT-549-sh, BT-549-NC, MDA-231-sh, and MDA-231-NC cells. The knockdown and negative control cells were treated with TGF-β (5 ng/ml) and inoculated in the upper chamber. Compared with the negative control cells, the invasion rate of BT-549-sh cells was 36.3%, and that of MDA-231-sh was 45.1% (Figure 2B). These data showed that *DCST1-AS1* promotes TGF-β-induced invasion potential of BT-549 and MDA-MD-231.

MMP2 and MMP9 play an important role in cancer metastasis by promoting the degradation of extracellular matrix (13). TGF-β can enhance the tumorigenicity and invasiveness of breast cancer cells by inducing the expression of MMP2 and MMP9 (14). Using ELISA, we determined the levels of MMP2 and MMP9 in the cell culture supernatants before and after TGF-β treatment by plotting a concentration standard curve. As shown in Figure 2C, prior to TGF-β induction, there was no significant change in MMP2 secretion by *DCST1-AS1* knockdown cells, but following 24 h of TGF-β induction, the level of MMP2 secreted by *DCST1-AS1* knockdown cells was significantly increased. Unlike MMP2, the level of MMP9 in *DCST1-AS1* knockdown cells was significantly decreased prior to and after TGF-β induction, indicating that the knockdown of *DCST1-AS1* affects the expression of MMP9. These data showed that *DCST1-AS1* affects TGF-β-induced secretion of MMP2 and MMP9 in BT-549 and MDA-MD-231 cells.

### *DCST1-AS1* Associates With ANXA1

**Figure 3:**
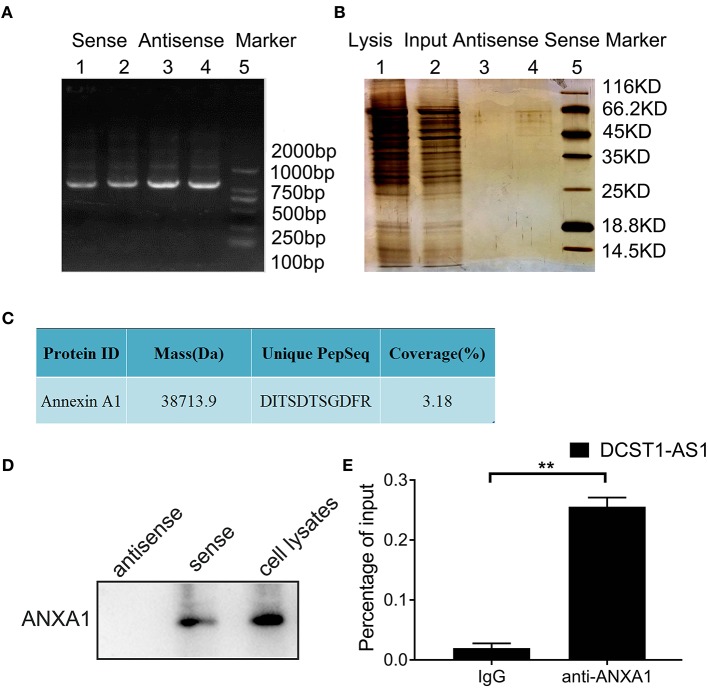
*DCST1-AS1* interacts with ANXA1 in BT-549 cells. **(A)** Gel electrophoresis of the PCR products of the sense and antisense strands of *DCST1-AS1*. **(B)** SDS-PAGE of proteins pulled down using the *DCST1-AS1* sense and antisense strands. **(C)** Analysis of the pulled-down proteins by liquid chromatography/tandem mass spectrometry **(D)** Western blotting was used to confirm the results of mass spectrometry. ANXA1 was detected in the proteins pulled down with the *DCST1-AS1* sense strand. Proteins pulled down with the *DCST1-AS1* antisense strand were used as a negative control, and whole-cell lysate was used as a positive control. **(E)** The RNA immunoprecipitation products were isolated and purified, and the amount of *DCST1-AS1* bound to ANXA1 or IgG was measured by RT-qPCR. IgG was used as a negative control. 18S rRNA was used as an internal control. **p < 0.01.

To identify the proteins that bind to *DCST1-AS1*, we performed RNA pulldown assays. First, the full-length plasmid of *DCST1-AS1*, pUC57-DCST1-AS1, was synthesized and fully sequenced. Then, using pUC57-DCST1-AS1 as a template, biotin-labeled sense and antisense *DCST1-AS1* were transcribed *in vitro*, and incubated with BT-549 cell lysate to form nucleic acid–protein complexes. The complexes capable of binding to streptavidin-labeled magnetic beads were separated from other components in the incubation solution and subjected to SDS-PAGE and silver staining (Figures 3A,B). Subsequently, the proteins that were pulled down using sense and antisense *DCST1-AS1* were treated with trypsin and analyzed by liquid chromatography/tandem mass spectrometry (LC-MS/MS). The mass spectrometry data were searched and analyzed using the Proteinpilot software (Supplementary Table). Using stringent parameters including confidence interval of ≥95% and unique peptides ≥1, the common contaminating proteins such as keratin, antibody proteins, and serum albumin were excluded, and we found that the sense *DCST1-AS1*, but not the antisense, specifically associated with ANXA1 in BT-549 cells (Figure 3C). We analyzed the remaining pulldown protein solution using Western blotting and found that ANXA1 was specifically detected in the protein solution pulled out using the sense *DCST1-AS1*, which is consistent with the mass spectrometry results (Figure 3D). We further verified the binding specificity between *DCST1-AS1* and ANXA1 using RNA immunoprecipitation, using ANXA1 antibody to precipitate RNA that binds to ANXA1 in BT-549 cell lysate. We isolated and purified the precipitate and successfully detected *DCST1-AS1* by RT-PCR amplification (Figure 3E). These experimental data demonstrated that *DCST1-AS1* stably binds to ANXA1 protein in BT-549 cells.

### *DCST1-AS1* Affects ANXA1 Expression

**Figure 4:**
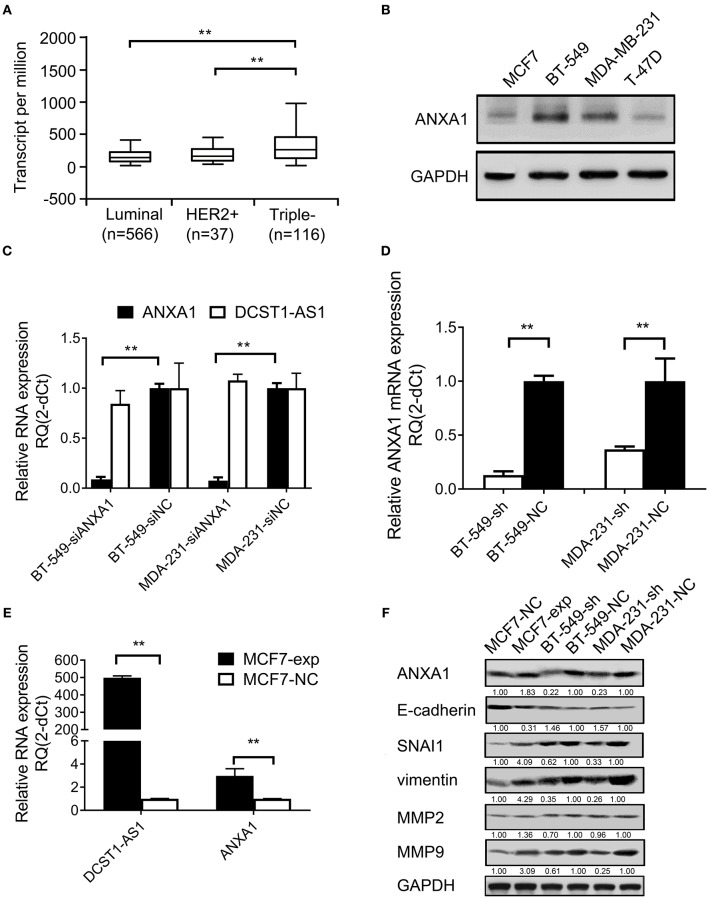
Effect of *DCST1-AS1* on the expression of ANXA1 in breast cancer cells. **(A)** TCGA data were used to analyze the expression of ANXA1 in the breast cancer subtypes. **(B)** Western blotting was used to analyze the expression of ANXA1 in BT-549, MDA-MB-231, MCF7, and T-47D cells. GAPDH was used as an internal control. **(C)** RT-PCR was used to analyze the changes in *DCST1-AS1* expression in BT-549 and MDA-MB-231 cells following knockdown of ANXA1. siANXA1, small interfering RNA targeting ANXA1. siNC, negative control siRNA. **(D)** RT-PCR was used to analyze the changes in ANXA1 mRNA expression in BT-549 and MDA-MB-231 cells following knockdown of DCST1-AS1. **(E)** RT-PCR was used to analyze ANXA1 mRNA expression following overexpression of *DCST1-AS1* in MCF7 cells. **(F)** Western blotting was used to analyze the effect of *DCST1-AS1* on expression of ANXA1, E-cadherin, vimentin, SNAI1, MMP9, and MMP2 in BT-549, MDA-MB-231, and MCF7 cells. **p < 0.01.

We analyzed TCGA database and found that *ANXA1* is expressed in several breast cancer subtypes (luminal, HER2+, and TNBC), which have different morphological, clinical, and therapeutic responses. We found that *ANXA1* is expressed significantly higher in the TNBC subtype than in the luminal and HER2+ types (Figure 4A). We used Western blotting to detect the expression level of ANXA1 in breast cancer cells (BT-549, MDA-MB-231, MCF7, and T-47D) and found that ANXA1 level was significantly higher in BT-549 and MDA-MB-231 cells (highly invasive) than in MCF7 and T-47D cells (low invasive) (Figure 4B), which was consistent with the expression of *DCST1-AS1* (11). We designed a siRNA targeting *ANXA1* and transfected BT-549 and MDA-MB-231 cells. We found that knockdown of *ANXA1* had no significant effect on the expression of *DCST1-AS1* (Figure 4C). Subsequently, we examined *ANXA1* mRNA in control and stable *DCST1-AS1* knockdown cell lines MDA-231-sh, MDA-231-NC, BT-549-sh, and BT-549-NC cells. *ANXA1* mRNA was downregulated in BT-549-sh and MDA-231-sh cells compared to the negative control group (Figure 4D). We transfected MCF7 cells (with low endogenous expression of *DCST1-AS1*) with *DCST1-AS1* overexpression lentiviral vector to obtain a cell line stably expressing *DCST1-AS1* and its corresponding negative control, labeled MCF7-exp and MCF7-NC, respectively. RT-PCR analysis showed that the upregulation of *DCST1-AS1* in MCF7-exp cells was accompanied by the upregulation of *ANXA1* mRNA (Figure 4E). Taken together, these results indicate that *DCST1-AS1* is involved in *ANXA1* mRNA regulation.

In addition, we examined ANXA1 and other key EMT-associated proteins and found that the expression of ANXA1, vimentin, SNAI1, and MMP9 were significantly decreased in BT-549 and MDA-MB-231 cells following *DCST1-AS1* knockdown, while the expression of E-cadherin was increased. Conversely, overexpression of *DCST1-AS1* in MCF7 reversed the expression pattern of these proteins (Figure 4F). We also performed *DCST1-AS1* overexpression experiments in MDA-MB-231 cells; however, the Western blotting showed no significant changes in ANXA1 (Supplementary Figure 2). We speculate that this is related to the high levels of endogenous expression of *DCST1-AS1* and ANXA1 in MDA-MB-231 cells. MMP2 protein does not appear to be affected by the amount of *DCST1-AS1* expression, which is consistent with our previous ELISA assay results.

### *DCST1-AS1* Promotes TGF-β/Smad-Induced EMT Through ANXA1

**Figure 5:**
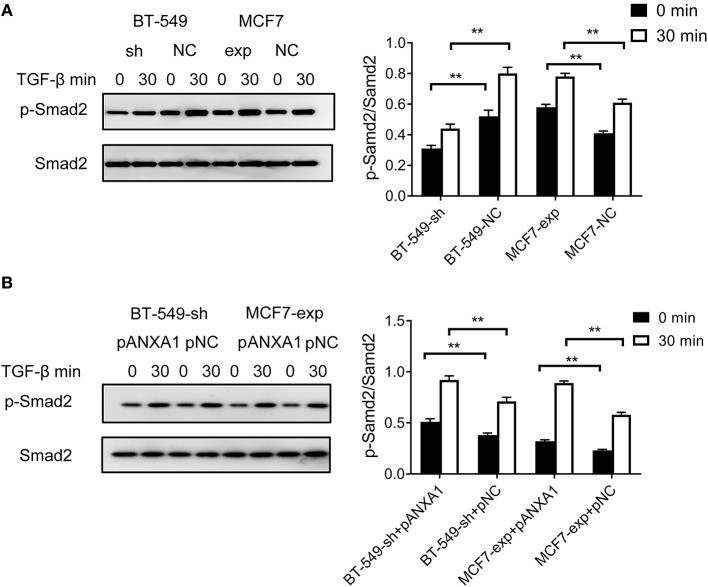
Effect of *DCST1-AS1* in combination with ANXA1 on TGF-β/Smad signaling. **(A)** Western blotting was used to analyze the effects of deletion and overexpression of *DCST1-AS1* on the ratio of p-Smad2 and Smad2 in BT-549 and MCF7 cells. **(B)** Western blotting was used to analyze the effect of exogenous expression of ANXA1 on the ratio of p-Smad2 and Smad2 in BT-549-sh and MCF7-exp cells. pANXA1, pcDNA3.1-ANXA1; pNC, negative control. **p < 0.01.

We treated BT-549-sh and BT-549-NC cells with 5 ng/ml of TGF-β. Compared to the negative control cells, we found that BT-549-sh cells exhibited impaired phosphorylation of Smad2 following TGF-β treatment. MCF7 cells endogenously express *DCST1-AS1* and ANXA1 at low levels. Based on this characteristic, we performed functional experiments with MCF7 cells: TGF-β was added to MCF7-exp and MCF7-NC cells. Compared with the control group, the phosphorylation of Smad2 in *DCST1-AS1-*overexpressing cells was increased to a certain extent (Figure 5A). Our results indicate that *DCST1-AS1* is involved in the regulation of TGF-β/Smad signaling in BT-549 and MCF7 cells.

Since knockdown of *DCST1-AS1* reduced the expression level of ANXA1 in BT-549 cells and the recombinant eukaryotic expression vector pcDNA3.1-ANXA1 effectively upregulates ANXA1 (Supplementary Figure 3), we designed an ANXA1 rescue experiment by transfecting pcDNA3.1-ANXA1 into BT-549-sh cells. After 48 h of transfection, the cells were induced with TGF-β for 30 min. We found that exogenous ANXA1 partially reversed the impaired Smad2 phosphorylation status caused by *DCST1-AS1* knockdown (Figure 5B). We also transfected the expression vector pcDNA3.1-ANXA1 in MCF7-exp cells and treated the cells with the same amount of TGF-β. We found that Smad2 phosphorylation levels were significantly increased in MCF7-exp cells that express exogenous ANXA1. Our results indicate that *DCST1-AS1* and ANXA1 promote EMT through TGF-β/Smad signaling and are not limited to TNBC cells alone.

### *DCST1-AS1* Enhances Cell Chemoresistance via ANXA1

**Figure 6:**
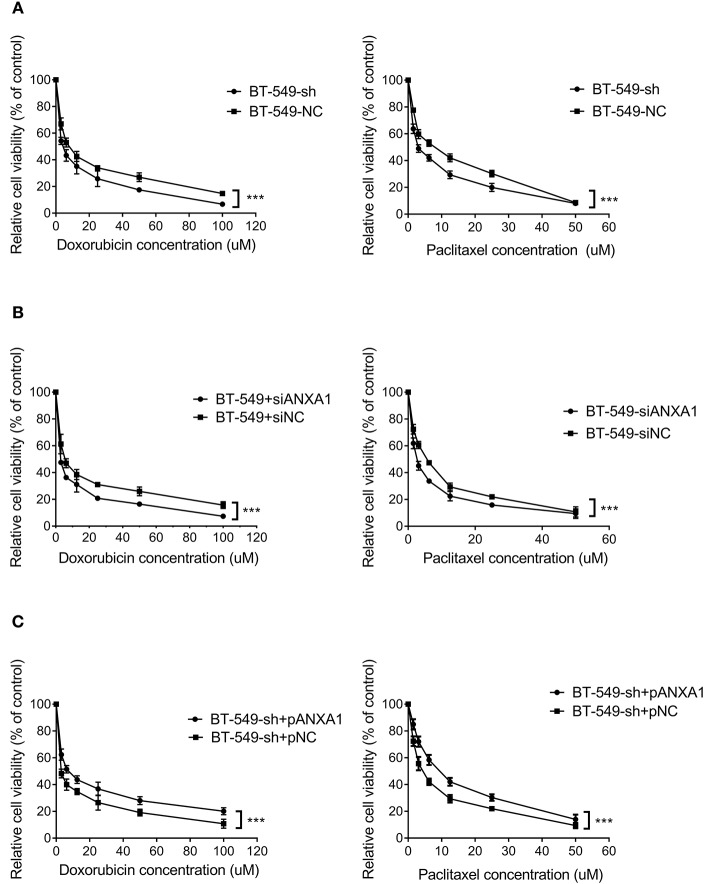
*DCST1-AS1* in combination with ANXA1 enhances chemoresistance in BT-549 cells. **(A)** The chemosensitivity of BT-549 cells to doxorubicin and paclitaxel following knockdown of DCST1-AS1 was analyzed using CCK8 assay. **(B)** The chemosensitivity of BT-549 cells to doxorubicin and paclitaxel following knockdown of ANXA1 was analyzed using CCK8 method. **(C)** The chemosensitivity of BT-549-sh cells to doxorubicin and paclitaxel following exogenous expression of ANXA1was analyzed using CCK8 assay. ***p < 0.001.

To clarify the therapeutic significance of *DCST1-AS1* in association with ANXA1 in TNBC, we chose doxorubicin and paclitaxel for the drug sensitivity experiments. The CCK-8 method was used to measure the cell inhibition rate, and the change in chemoresistance of cells was evaluated by calculating the half maximal inhibitory concentration (IC_50_) in combination with the quantitative response curve. We first performed loss of function assays. After treating BT-549-sh and BT-549-NC cells with different concentrations of doxorubicin and paclitaxel for 48 h, we observed that the IC_50_ values of the *DCST1-AS1* knockdown cells were significantly lower than those of the negative control cells, indicating that *DCST1-AS1* expression favored BT-549 resistance to doxorubicin and paclitaxel (Figure 6A). In BT-549 cells with knockdown of ANXA1, we observed that the IC_50_ of the doxorubicin group decreased significantly from 5.98 ± 0.14 to 2.70 ± 0.08, and that of the paclitaxel group decreased significantly from 5.07 ± 0.14 to 2.66 ± 0.08, indicating that ANXA1 mediates BT-549 resistance to doxorubicin and paclitaxel (Figure 6B). We have demonstrated that knockdown of *DCST1-AS1* is accompanied by a decrease in the amount of ANXA1 expression. Based on this, we designed a rescue experiment by transfecting pcDNA3.1-ANXA1 into *DCST1-AS1* knockdown cells to restore ANXA1 expression, and then subjected the transfected cells to doxorubicin and paclitaxel treatment. We found that exogenous expression of ANXA1 restored the loss of resistance of BT-549 cells due to *DCST1-AS1* knockdown. In the doxorubicin group, the IC_50_ increased significantly from 3.03 ± 0.27 to 7.81 ± 0.31, and in the paclitaxel group, the IC_50_ increased significantly from 4.43 ± 0.15 to 9.01 ± 0.25 (Figure 6C). We have summarized the IC_50_ values from different experiments in Table 1. Taken together, the experimental data from this study demonstrate that *DCST1-AS1* is involved in the regulation of drug resistance of BT-549 cells to doxorubicin and paclitaxel by binding to ANXA1.

**Table 1:** The effect of *DCST1-AS1* and ANXA1 on IC_50_ values when BT-549 cells were treated with doxorubicin and paclitaxel.

**Group**	**IC**_****50****_ **value (μM mean** **±** **SD)**			
	**Doxorubicin**	***P***	**Paclitaxel**	***p***
BT-549-sh	4.33 ± 0.30	<0.001	3.40 ± 0.18	<0.001
BT-549-NC	8.43 ± 0.37		6.78 ± 0.50	
BT-549-siANXA1	2.70 ± 0.08	<0.001	2.66 ± 0.08	<0.001
BT-549-siNC	5.98 ± 0.14		5.07 ± 0.14	
BT-549-sh+pANXA1	7.81 ± 0.31	<0.001	9.01 ± 0.25	<0.001
BT-549-sh+pNC	3.03 ± 0.27		4.43 ± 0.15	

## Discussion

EMT is an important event in cancer metastasis (15). During this process, epithelial cells lose their epithelial characteristics and acquire more mesenchymal properties through cytoskeletal rearrangement, adhesion, and changes in cell structure and morphology. TGF-β, a major inducer of EMT, is overexpressed in breast cancer, and is associated with malignant progression and poor prognosis (16). Adding TGF-β to epithelial cells in culture induces EMT *in vitro* (17, 18). TGF-β induces EMT through a Smad-dependent pathway. Briefly, TGF-β binds to the type II receptor (TGFR2) and phosphorylates the type I receptor (TGFR1), which in turn induces phosphorylation of the Smad protein to control transcription of the target genes. TGF-β also enhances EMT through Smad-independent pathways, such as through small GTPase, ERK1/2, and p38 MAPK pathways (19). These pathways overlap and collectively regulate EMT-related transcription factors and act as major molecular switches in the EMT program.

Downregulation of E-cadherin and upregulation of vimentin are hallmarks of EMT (20). Inhibition of E-cadherin expression by transcription factors is central to the progression of EMT. E-cadherin is an important regulator of the epithelial phenotype, and changes in its expression levels can lead to changes in the motility and invasiveness of tumor cells. The level of E-cadherin is inversely related to the survival and progression in breast cancer patients (21). SNAI1 is an important EMT transcription factor that regulates EMT progression by inhibiting the migration of E-cadherin and enhancing the migration and invasion of tumor cells (22). Vimentin belongs to the type III intermediate filament, which is usually expressed in mesenchymal cells and some ectodermal cells, such as fibroblasts and endothelial cells. Blocking the expression of vimentin can re-epithelialize cells that have undergone EMT and can attenuate the invasive ability of tumor cells (23). By examining changes in these important EMT-related indicators, we confirmed that *DCST1-AS1* was involved in the EMT process in breast cancer.

The EMT process not only changes the cellular characteristics of cancer cells from an epithelial to mesenchymal phenotype, but also triggers remodeling of the extracellular matrix by activating various proteases such as the MMPs, thereby achieving anoikis resistance, and increased invasiveness and migration (24). MMPs are a group of enzymes that regulate the extracellular matrix. Among them, the metalloproteinases MMP2 and MMP9 that play a key role in the degradation of type IV collagen are overexpressed in breast cancer and correlate with poor prognosis of patients (25). TGF-β/Smad signaling can upregulate MMP2 and MMP9 to induce breast cancer cell invasion (26). Our study confirmed that disruption of *DCST1-AS1* in BT-549 and MDA-MD-231 cells affected TGF-β-induced secretion of MMP2 and MMP9 in cell culture supernatants. Furthermore, we found that MMP9 was more sensitive to changes in *DCST1-AS1* than MMP2. Given that ANXA1 positively regulates the expression and function of MMP9 in invasive breast cancer (27), we believe that the sensitivity of MMP9 may be related to the effect of *DCST1-AS1* on ANXA1.

The overexpression of ANXA1 in highly aggressive breast cancers has led many researchers to focus on its role in invasion and metastasis. Swa et al. cultured normal breast epithelial cells from ANXA1-hybrid and ANXA1-deficient mice, and showed that ANXA1 may play a role in cell adhesion and migration pathways (28). Alli-Shaik et al. analyzed mammary epithelial cells from ANXA1-hybrid and ANXA1-deficient mice by quantitative phosphorylation proteomics and found that most ANXA1-responsive phosphorylation changes occurred in proteins involved in cytoskeletal reorganization, which favors breast cancer cell migration (29). Bist et al. demonstrated that ANXA1 interacts with NEMO and RIP1 to constitutively activate IKK complexes and NF-κB, promoting breast cancer invasion and metastasis (30). Overexpression of ANXA1 is often associated with EMT in some aggressive cancers (31, 32). TGF-β/Smad signaling plays an important role in the progression of EMT in breast cancer, in which phosphorylation of Smad2 triggers the formation of a complex between Smad2 and Smad4, resulting in nuclear translocation of the Smad2/Smad4 complex and subsequent gene transcription (12, 33, 34). In our study, the disruption of *DCST1-AS1* expression in BT-549 cells significantly reduced TGF-β-induced smad2 phosphorylation, while the overexpression of ANXA1 reversed this phenomenon. The results of overexpression of *DCST1-AS1* and ANXA1 in MCF7 cells not only confirmed our conclusion from the BT-549 cells, but also showed that EMT progression promoted by the binding of *DCST1-AS1* to ANXA1 through TGF-β/Smad signaling is not limited to TNBC cells. In addition, *DCST1-AS1* is closely related to ANXA1 in expression level and molecular function and is involved in regulating *ANXA1* mRNA. We believe that *DCST1-AS1* and ANXA1 are physically related, but our current research has not determined the binding site and whether this binding is assisted by other molecules. Cross-linked immunoprecipitation and high-throughput sequencing data from the ENCODE database indicate that IGF2BP1 binds to *ANXA1* mRNA. IGF2BP1 is known to be an RNA-binding protein that increases mRNA stability (35). We have shown that *DCST1-AS1* is involved in the regulation of IGF2BP1, so we speculate that the regulation of *ANXA1* mRNA by *DCST1-AS1* is related to IGF2BP1.

The resistance of tumor cells to anti-tumor drugs is a common cause for the failure of treatment in TNBC. The overexpression of several lncRNAs has been found to play a role in chemoresistance of TNBC. For example, lncRNA NEAT1 is overexpressed in TNBC cells that are resistant to chemotherapy (36). LncRNA HCP5 promotes cisplatin resistance in TNBC by regulating PTEN expression (37). LncRNA H19 confers chemoresistance to paclitaxel in TNBC cells by regulating the AKT signaling pathway (38). LncRNAs HIF1A-AS2 and AK124454 enhance the resistance of TNBC cells to paclitaxel (39). Upregulation of ANXA1 is associated with increased resistance to chemotherapy in several cancers, but the mechanism by which it promotes resistance is unclear. Jia et al. found that knocking down ANXA1 enhanced the chemosensitivity of multiple myeloma cells to bortezomib (40). Onozawa et al. found that overexpression of ANXA1 is associated with increased 5-FU resistance in colon cancer cells (41). Wang et al. detected increased expression of ANXA1 mRNA and protein in cisplatin-resistant lung cancer A549 cells and lung adenocarcinoma tissues (42). Studies in breast cancer have found that in the absence of detectable levels of P-glycoprotein and the breast cancer resistance protein, ANXA1 upregulation is associated with increased resistance to several anticancer drugs, including doxorubicin, melphalan, and etoposide (43). Researchers have found that upregulated ANXA1 is involved in the paclitaxel resistance in ovarian cancer cells (44). Anthracyclines and taxanes are the preferred cytotoxic drugs in TNBC chemotherapy. Doxorubicin blocks the DNA double-helix closure by inhibiting the DNA supercoiled topoisomerase II and disrupting DNA strand replication. Paclitaxel inhibits the normal dynamic regeneration of the microtubule network necessary for mitosis, prevents the formation of normal mitotic spindles, leads to chromosome breakage, and inhibits cell replication. Our study demonstrates that *DCST1-AS1* increases the resistance of BT-549 cells to doxorubicin and paclitaxel through ANXA1, thereby revealing the potential of *DCST1-AS1* in the clinical treatment of TNBC.

Deregulation of lncRNAs is often observed in various cancers and therefore lncRNAs have been proposed as predictive, diagnostic, and prognostic biomarkers, and as putative therapeutic targets (45, 46). Although several chemotherapeutic agents are currently used in the treatment of various cancers, including breast cancer, they have many side effects. The use of non-coding RNAs as therapeutic targets for breast cancer may provide a promising solution to these problems (47, 48). Tumor cells with mesenchymal differentiation often exhibit primary drug resistance, and EMT is considered to be an important mechanism through which tumor cells acquire drug resistance (49, 50). Our previous studies confirmed that *DCST1-AS1* promotes breast cancer cell proliferation, and knockdown of *DCST1-AS1* causes G2 phase arrest (11). In this study, we sought to identify the molecular pathways through which *DCST1-AS1* promotes breast cancer cell migration and invasion. Taken together, we demonstrate for the first time that *DCST1-AS1* promotes TGF-β-induced EMT and enhances doxorubicin and paclitaxel resistance in TNBC cells through ANXA1, revealing that *DCST1-AS1* may be a potential therapeutic target in TNBC.

## Data Availability Statement

All datasets generated for this study are included in the article/Supplementary Material.

## Author Contributions

LT contributed research design and drafted the manuscript. YC, PJ, and HC conducted experiments. LY, XT, and DM performed the data analyses. FY directed the project. All authors contributed to manuscript revision, and read and approved the submitted version.

### Conflict of Interest

The authors declare that the research was conducted in the absence of any commercial or financial relationships that could be construed as a potential conflict of interest.
