# Supplementary material for: DCST1-AS1 Promotes TGF-β-Induced Epithelial–Mesenchymal Transition and Enhances Chemoresistance in Triple-Negative Breast Cancer Cells via ANXA1
Source: Front Oncol. 2020 Mar 12;10:280. doi: 10.3389/fonc.2020.00280 (PMC7080863; doi:10.3389/fonc.2020.00280)
Supplement: Supplementary file 2 [file Data_Sheet_1.PDF]

Supplementary Figures

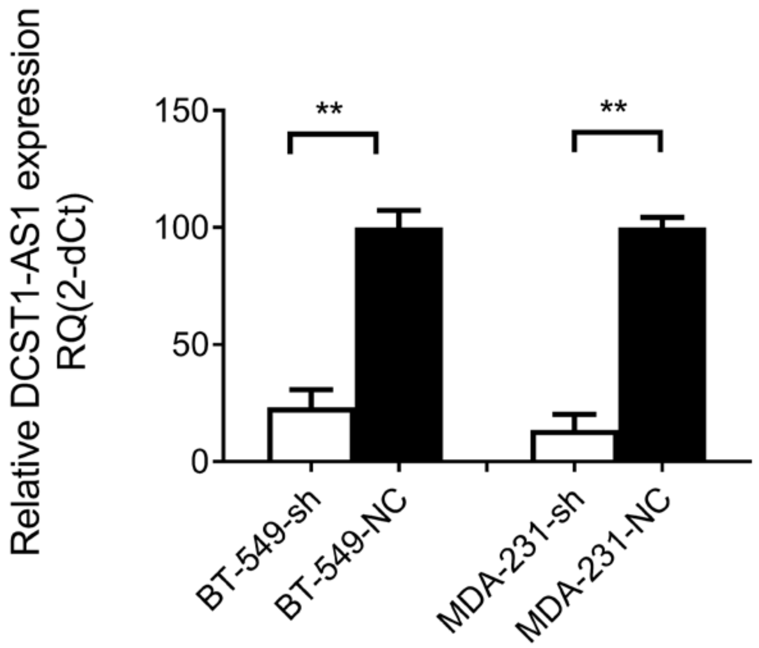

Figure S1. RT-PCR was used to analyze the efficiency of knockdown of *DCST1-AS1* in cells. **$p <$ 0.01.

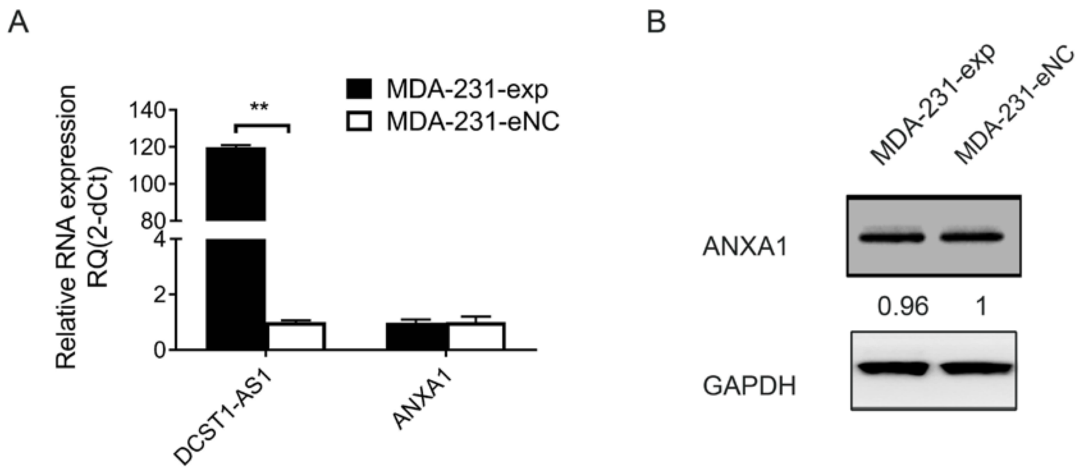

Figure S2. Effect of overexpression of *DCST1-AS1* on *ANXA1* expression. **(A)** RT-PCR was used to analyze the effect of overexpression of *DCST1-AS1* on *ANXA1* mRNA expression in MDA-MB-231 cells. **$p < 0.01$. **(B)** Western blotting was used to analyze the effect of overexpression of *DCST1-AS1* on ANXA1 protein expression in MDA-MB-231 cells.

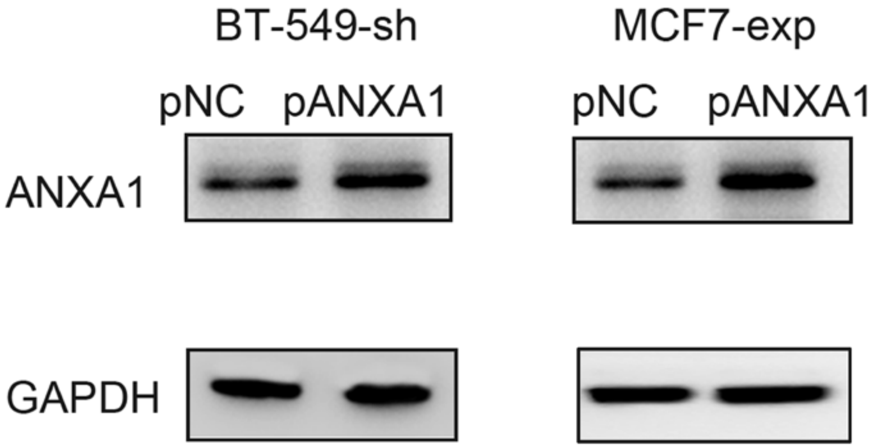

Figure S3. Western blot was used to analyze the change of ANXA1 protein expression in cells after transfection of the overexpression vector.
