# Supplementary figures and images for: DCST1-AS1 Promotes TGF-β-Induced Epithelial–Mesenchymal Transition and Enhances Chemoresistance in Triple-Negative Breast Cancer Cells via ANXA1
Source: Front Oncol. 2020 Mar 12;10:280. doi: 10.3389/fonc.2020.00280 (PMC7080863; doi:10.3389/fonc.2020.00280)

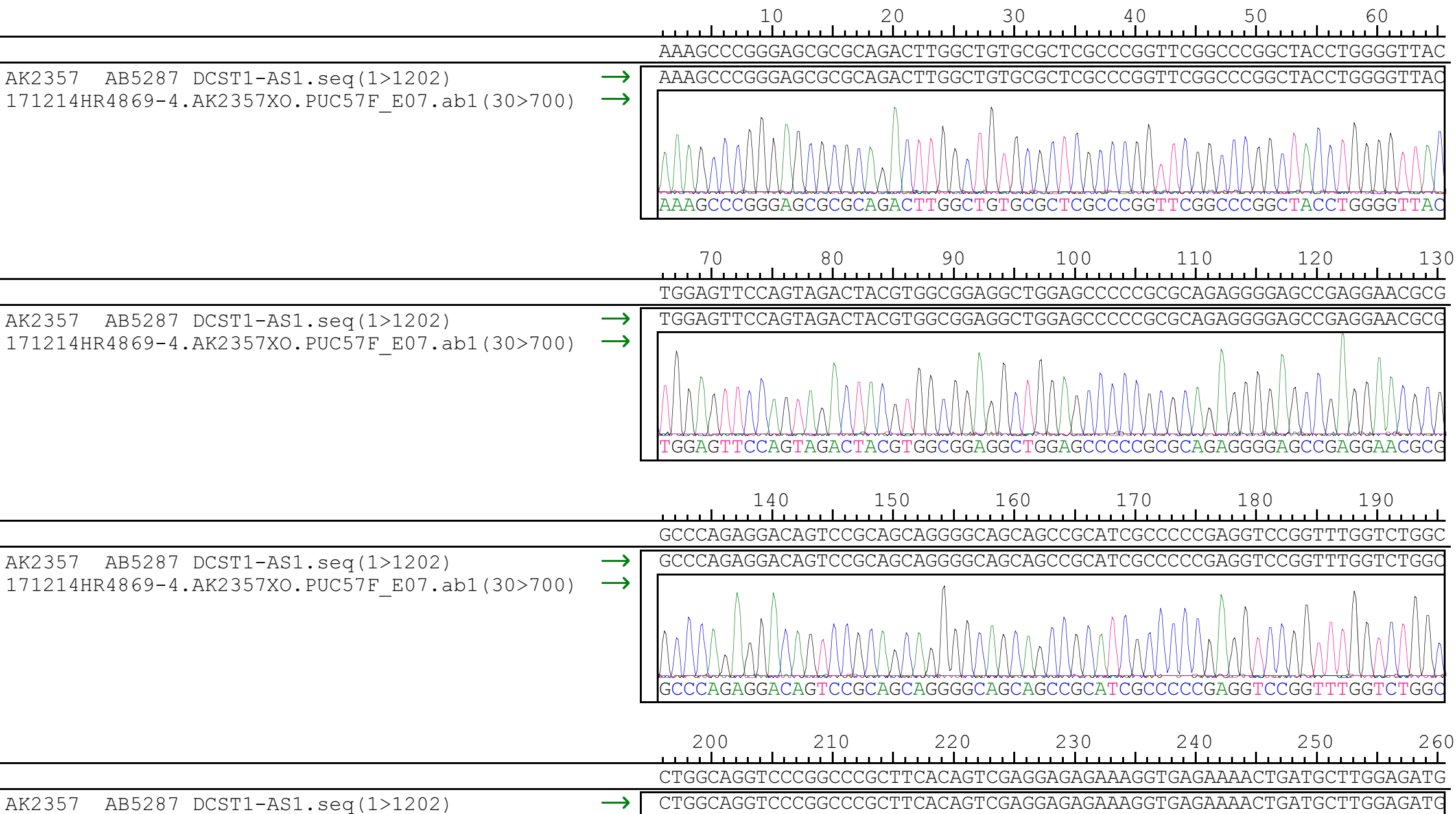

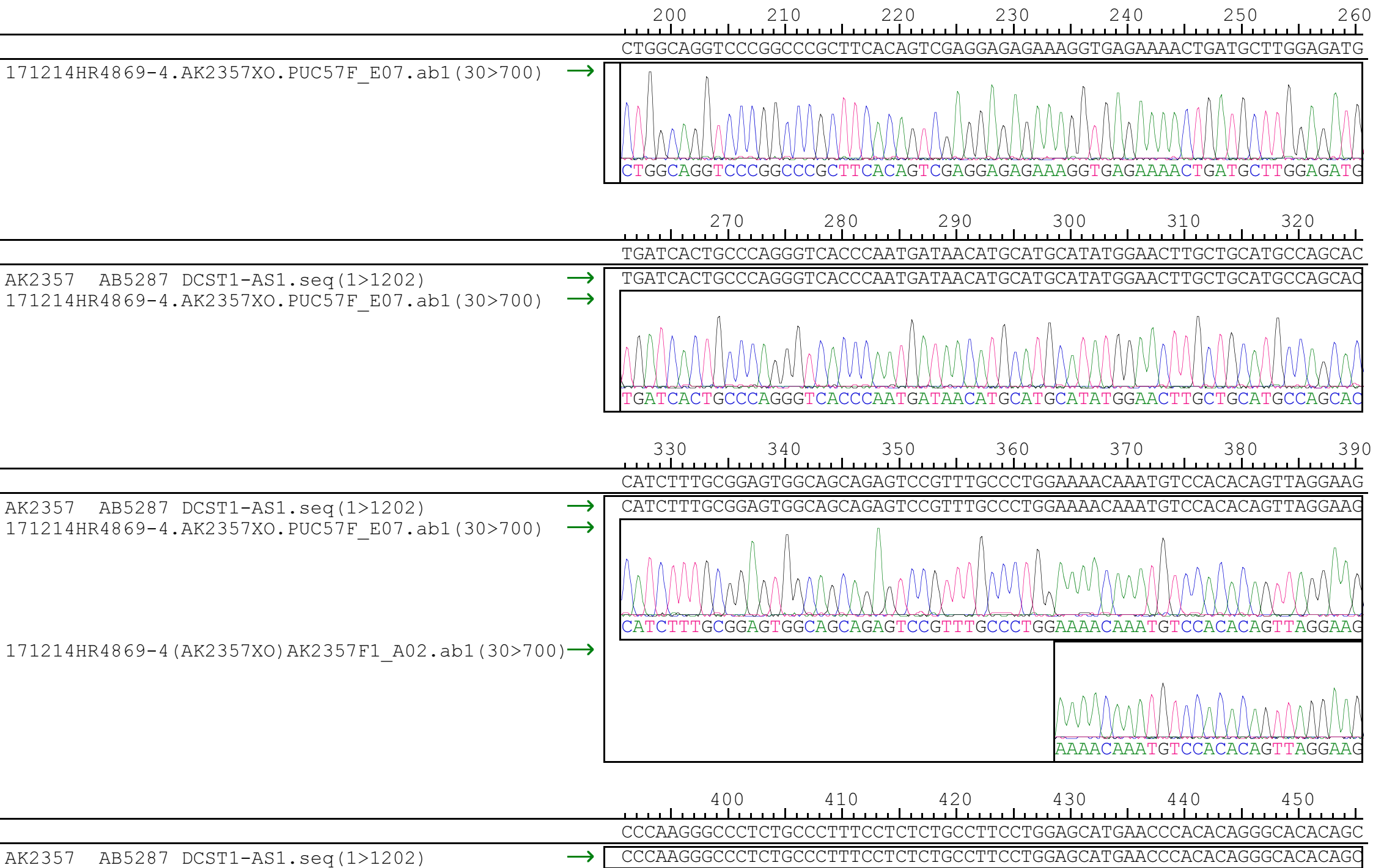

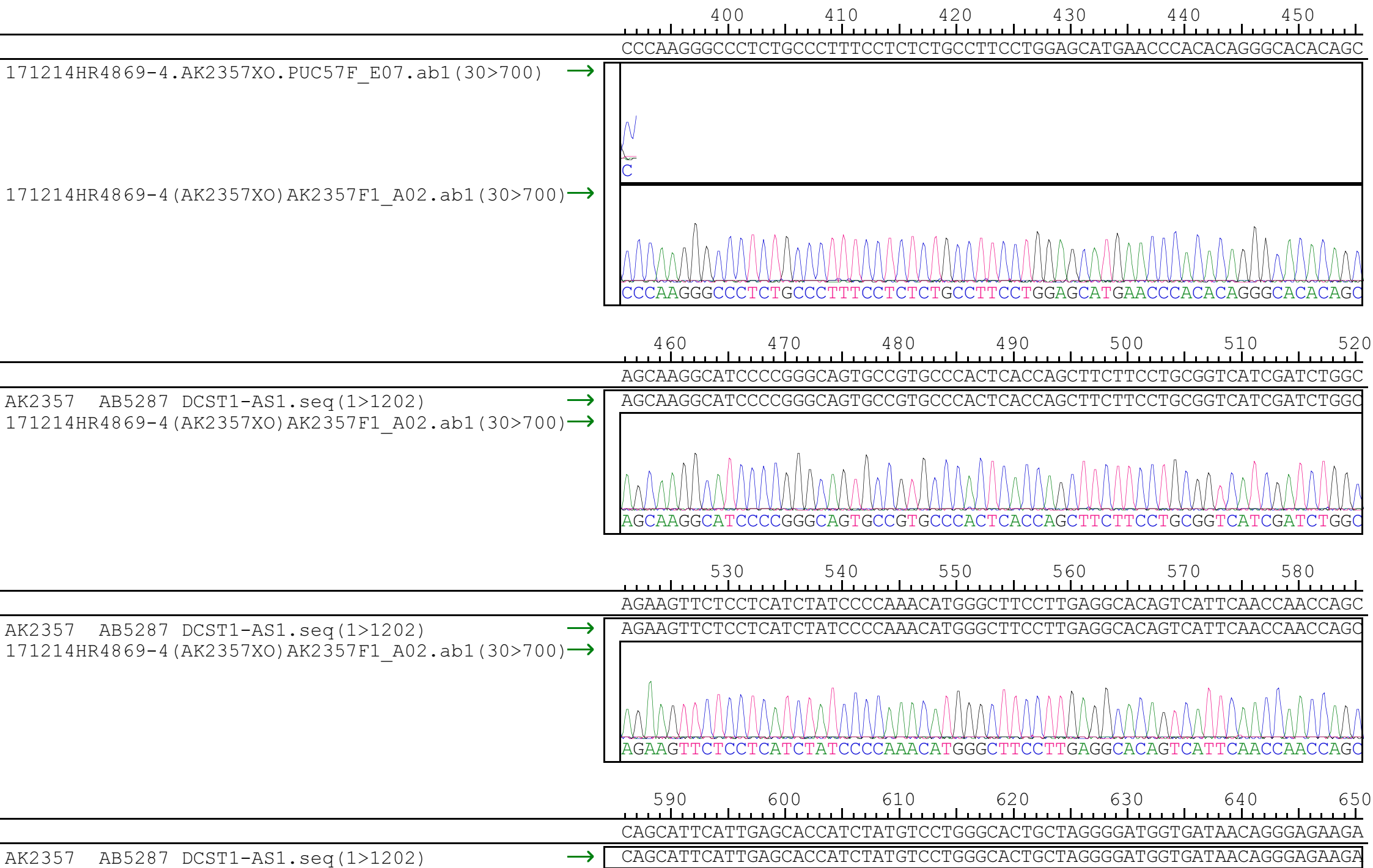

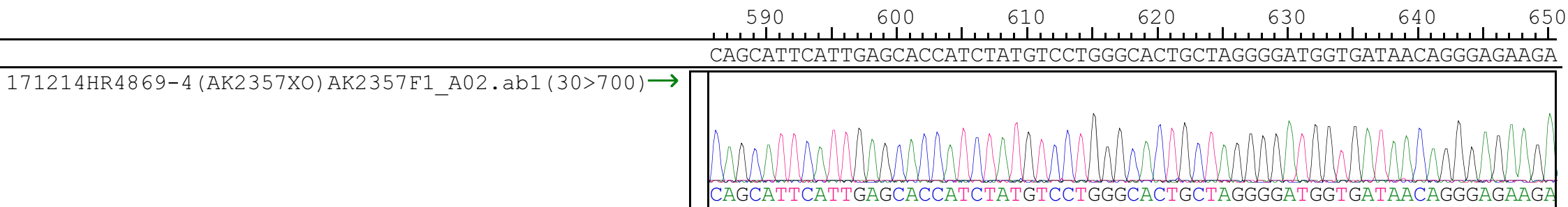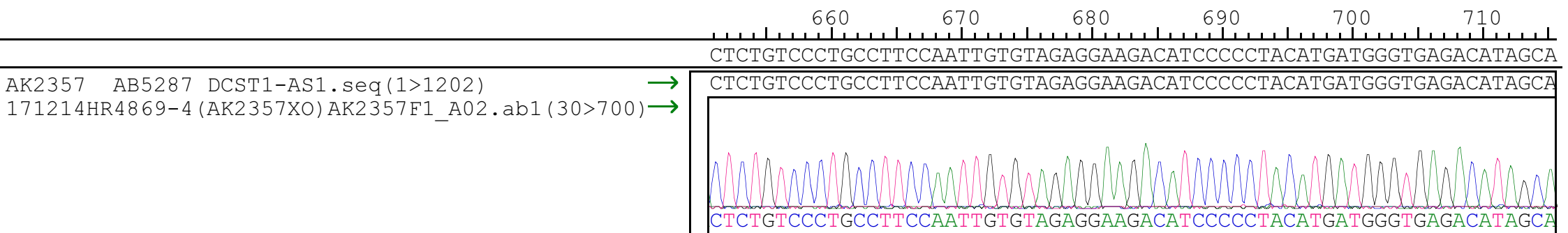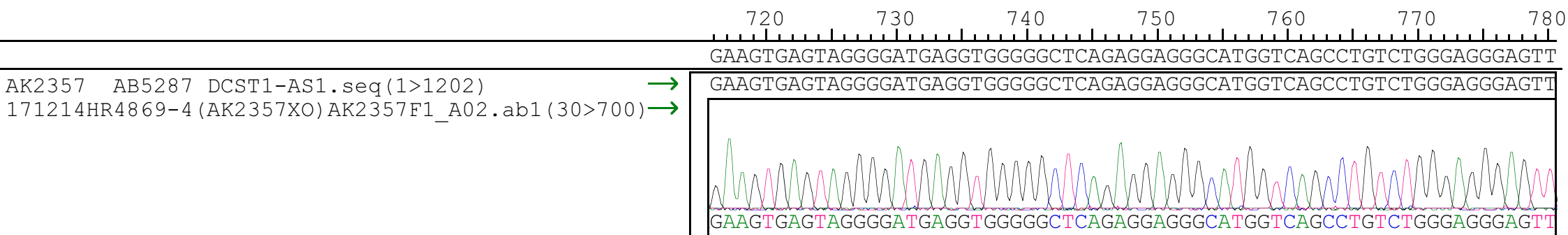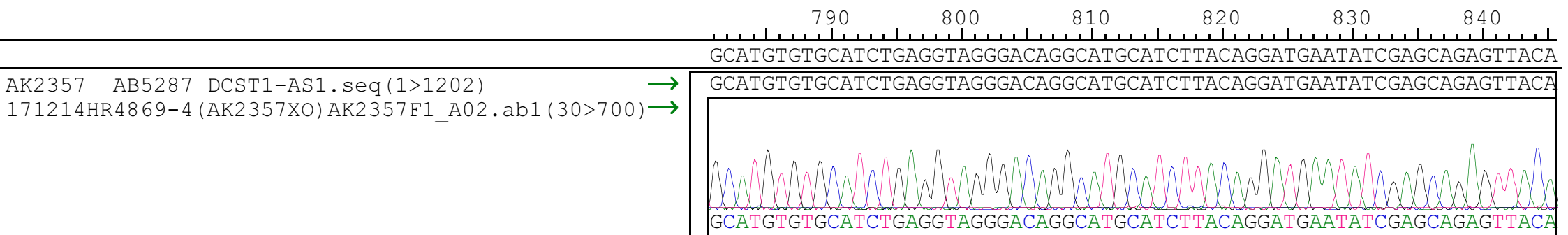

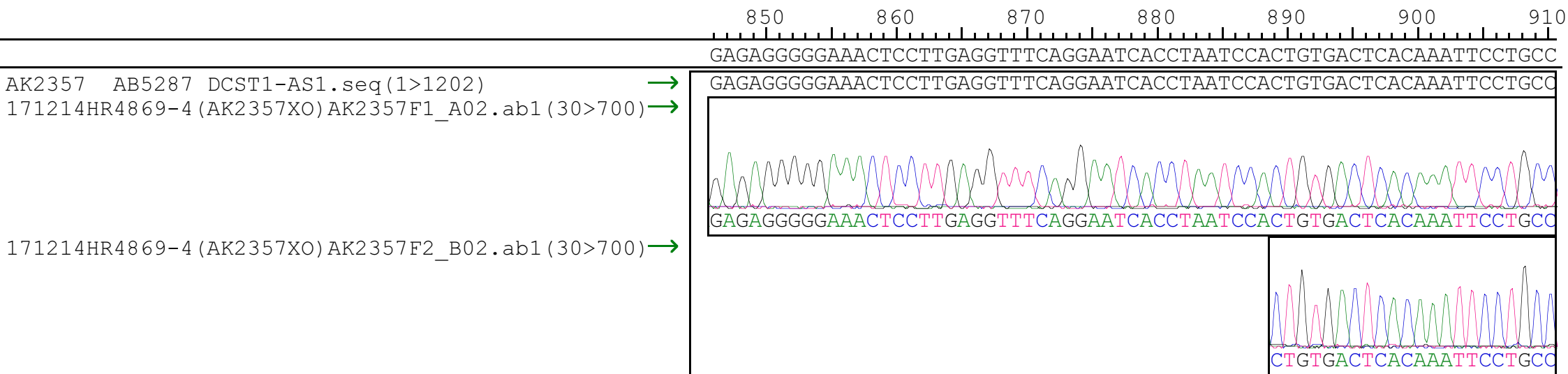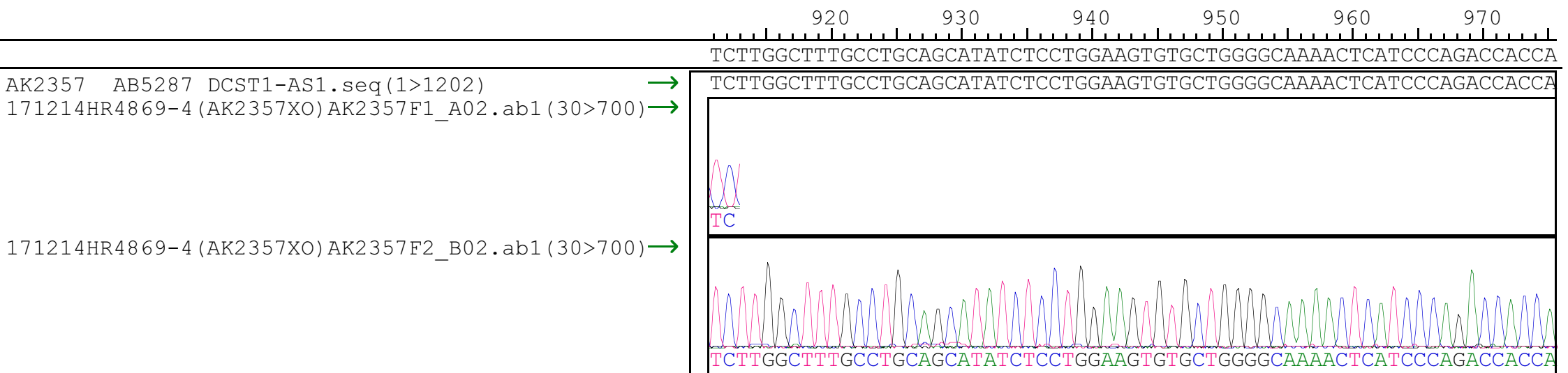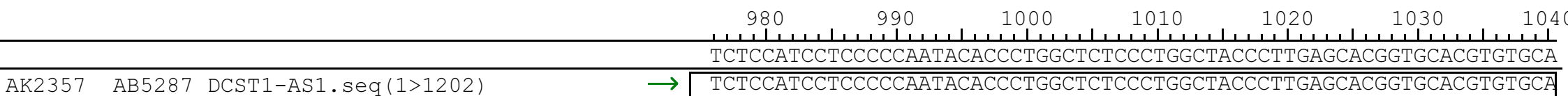

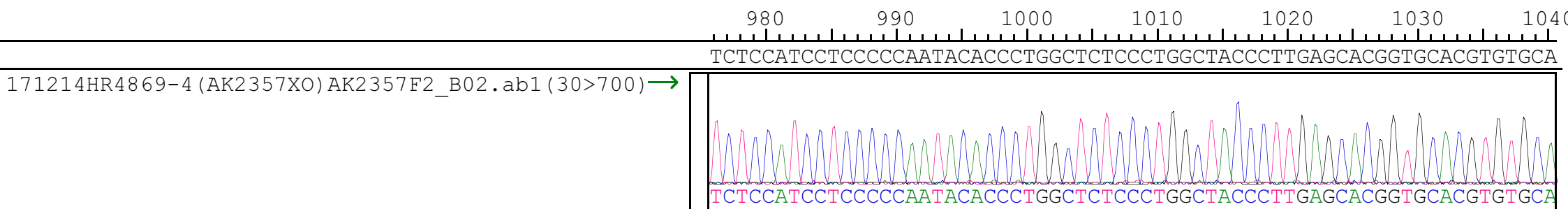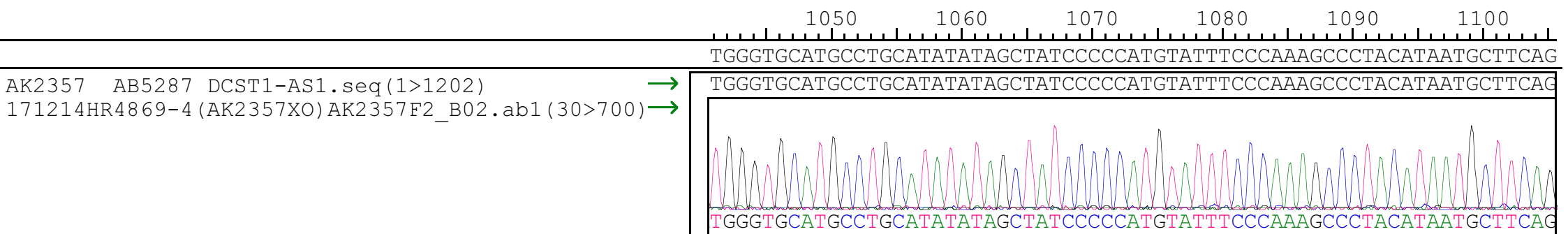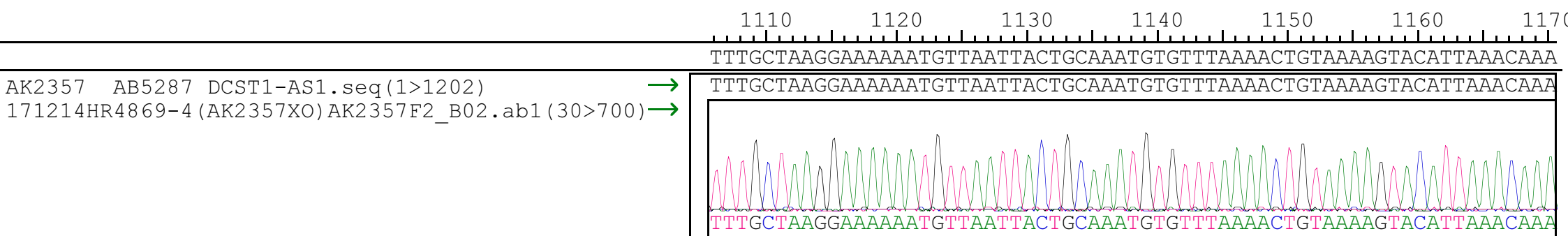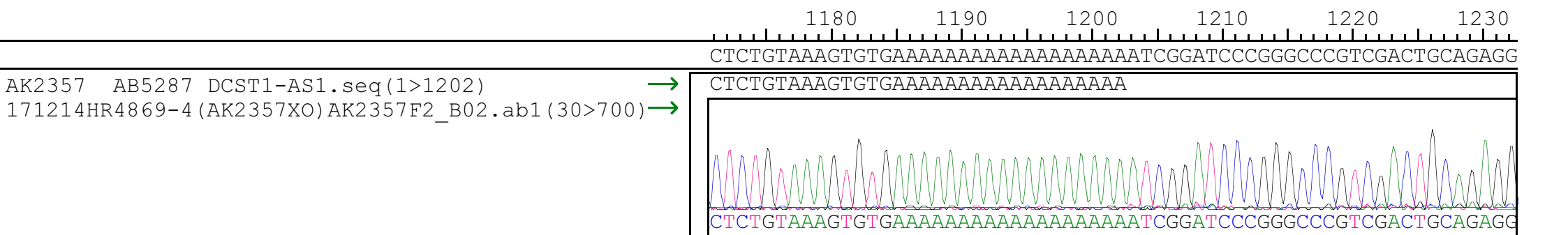

Supplement: Supplementary file 3 [file Data_Sheet_2.PDF]
