# Supplementary material for: DCST1-AS1 Promotes TGF-β-Induced Epithelial–Mesenchymal Transition and Enhances Chemoresistance in Triple-Negative Breast Cancer Cells via ANXA1
Source: Front Oncol. 2020 Mar 12;10:280. doi: 10.3389/fonc.2020.00280 (PMC7080863; doi:10.3389/fonc.2020.00280)
Supplement: Supplementary file 4 [file Data_Sheet_3.PDF]

10 20 30 40 50 60 70 80

TATAGGGAGACCCAAGCTGGCTAGCGTTTAAACTTAAGCTTGGTACCGAGCTCGGATCCGCCACCATGGCAATGGTATCAGAA

A5089-1_CMV-F_E08.ab1(50>500) →

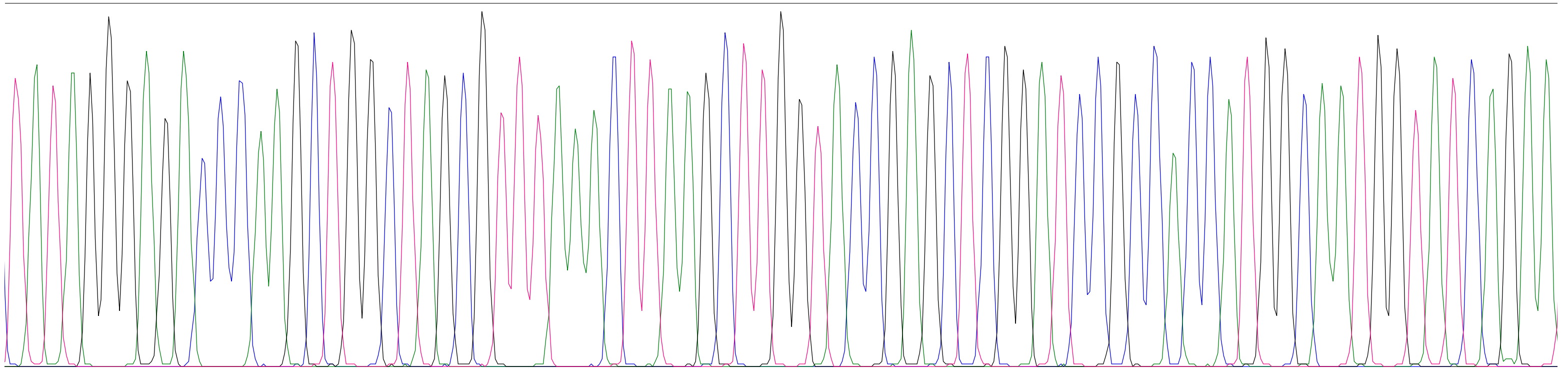

TATAGGGAGACCCAAGCTGGCTAGCGTTTAAACTTAAGCTTGGTACCGAGCTCGGATCCGCCACCATGGCAATGGTATCAGAA

B5081 Homo ANXA1.seq(1>1041) → ATGGCAATGGTATCAGAA

90 100 110 120 130 140 150 160

TTCCTCAAGCAGGCCTGGTTTATTGAAAATGAAGAGCAGGAATATGTTCAAACTGTGAAGTCATCCAAAGGTGGTCCCGGATC

A5089-1_CMV-F_E08.ab1(50>500) →

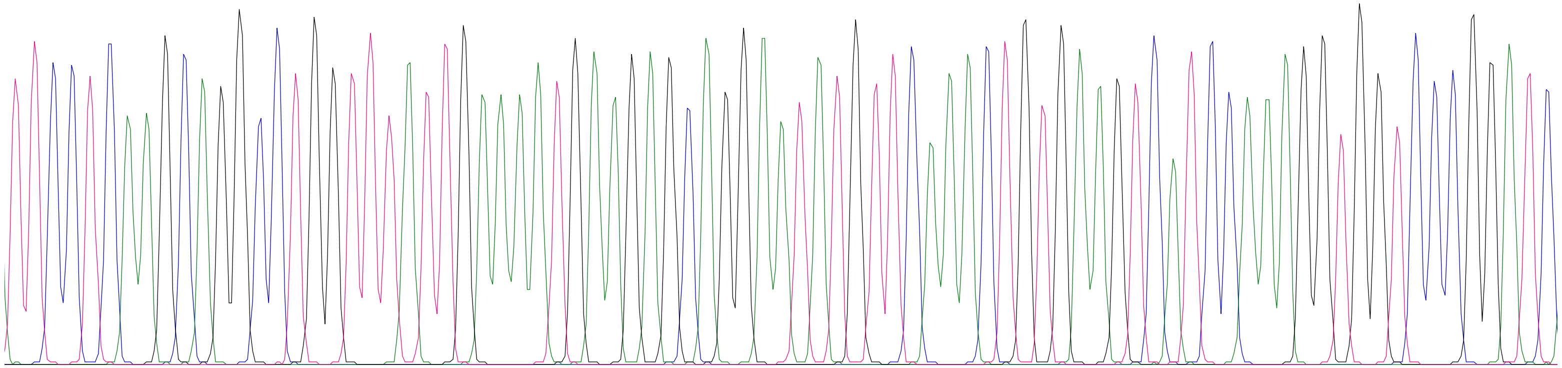

TTCCTCAAGCAGGCCTGGTTTATTGAAAATGAAGAGCAGGAATATGTTCAAACTGTGAAGTCATCCAAAGGTGGTCCCGGATC

B5081 Homo ANXA1.seq(1>1041) → TTCCTCAAGCAGGCCTGGTTTATTGAAAATGAAGAGCAGGAATATGTTCAAACTGTGAAGTCATCCAAAGGTGGTCCCGGATC

170 180 190 200 210 220 230 240

AGCGGTGAGCCCCTATCCTACCTTCAATCCATCCTCGGATGTCGCTGCCTTGCATAAGGCCATAATGGTTAAAGGTGTGGATG

```
                 170       180       190       200       210       220       230       240
                 AGCGGTGAGCCCCTATCCTACCTTCAATCCATCCTCGGATGTCGCTGCCTTGCATAAGGCCATAATGGTTAAAGGTGTGGATG
A5089-1_CMV-F_E08.ab1(50>500)  →
```

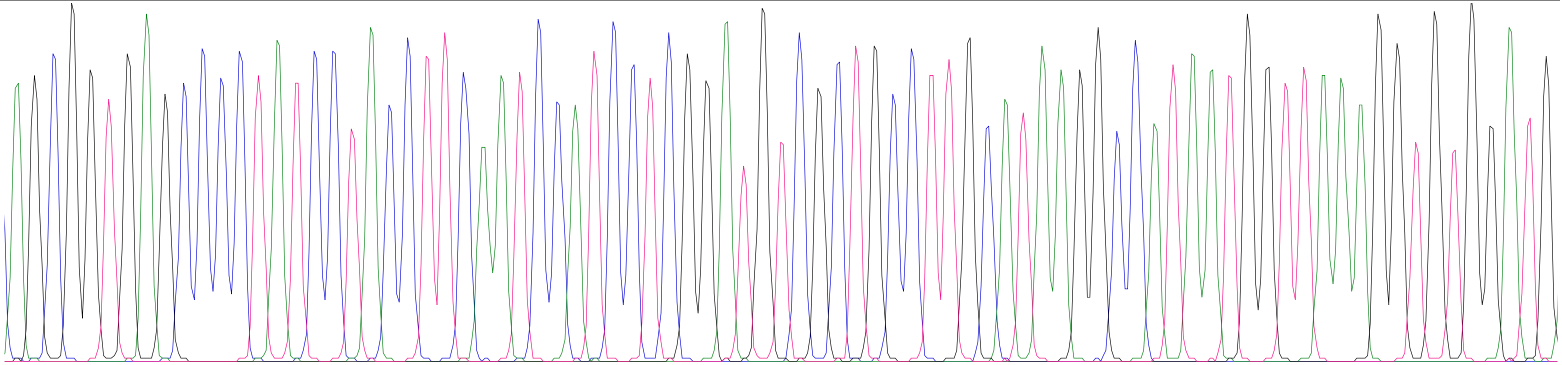

```
                 AGCGGTGAGCCCCTATCCTACCTTCAATCCATCCTCGGATGTCGCTGCCTTGCATAAGGCCATAATGGTTAAAGGTGTGGATG
B5081 Homo ANXA1.seq(1>1041)  →  AGCGGTGAGCCCCTATCCTACCTTCAATCCATCCTCGGATGTCGCTGCCTTGCATAAGGCCATAATGGTTAAAGGTGTGGATG
```

```
               250       260       270       280       290       300       310       320       330
                 AAGCAACCATCATTGACATTCTAACTAAGCGAAACAATGCACAGCGTCAACAGATCAAAGCAGCATATCTCCAGGAAACAGGA
A5089-1_CMV-F_E08.ab1(50>500)  →
```

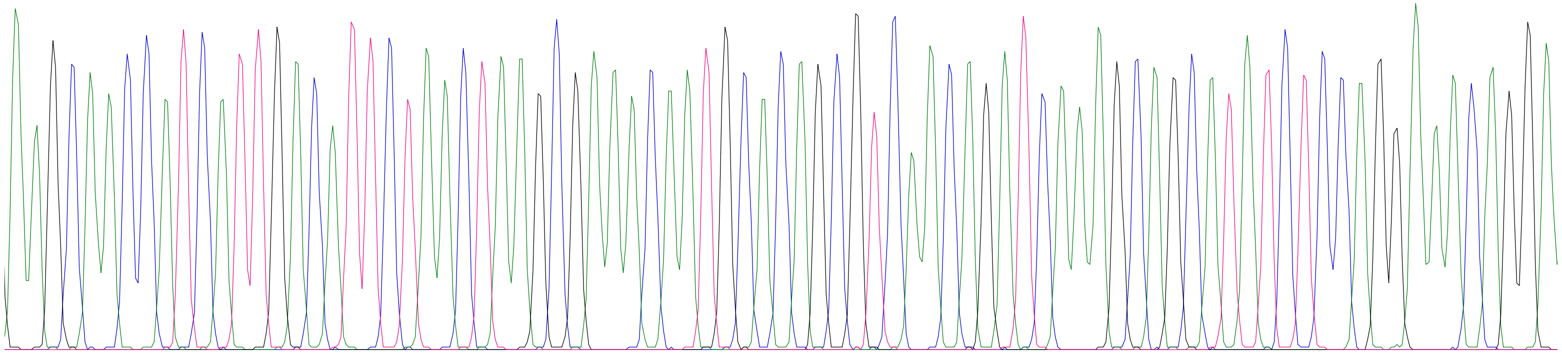

```
                 AAGCAACCATCATTGACATTCTAACTAAGCGAAACAATGCACAGCGTCAACAGATCAAAGCAGCATATCTCCAGGAAACAGGA
B5081 Homo ANXA1.seq(1>1041)  →  AAGCAACCATCATTGACATTCTAACTAAGCGAAACAATGCACAGCGTCAACAGATCAAAGCAGCATATCTCCAGGAAACAGGA
```

```
                    340       350       360       370       380       390       400       410
                 AAGCCCCTGGATGAAACACTGAAGAAAGCCCTTACAGGTCACCTTGAGGAGGTTGTTTTAGCTCTGCTAAAAACTCCAGCGCA
```

```
                                    340       350       360       370       380       390       400       410
                                 AAGCCCCTGGATGAAACACTGAAGAAAGCCCTTACAGGTCACCTTGAGGAGGTTGTTTTAGCTCTGCTAAAAACTCCAGCGCA
A5089-1_CMV-F_E08.ab1(50>500) →
```

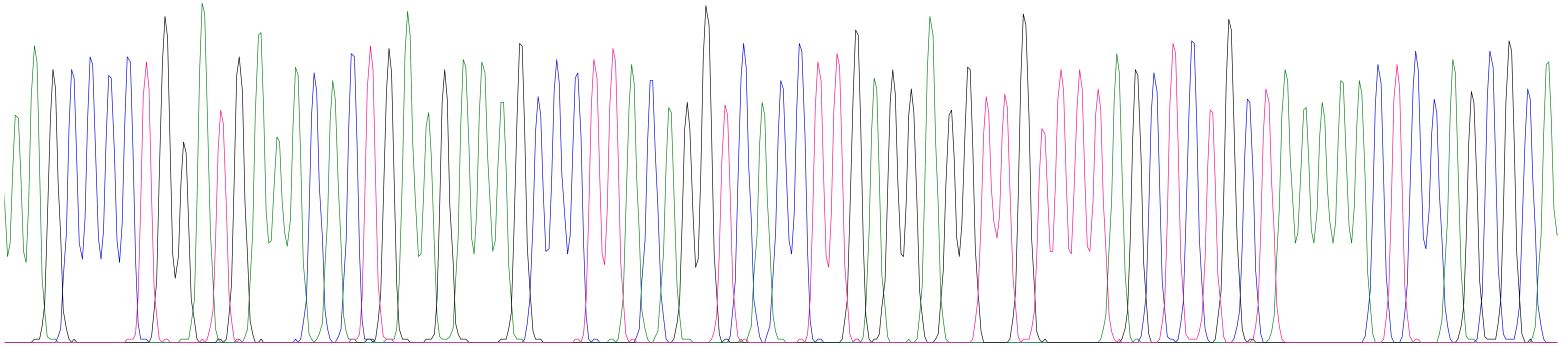

```
                                 AAGCCCCTGGATGAAACACTGAAGAAAGCCCTTACAGGTCACCTTGAGGAGGTTGTTTTAGCTCTGCTAAAAACTCCAGCGCA
B5081 Homo ANXA1.seq(1>1041)  →  AAGCCCCTGGATGAAACACTGAAGAAAGCCCTTACAGGTCACCTTGAGGAGGTTGTTTTAGCTCTGCTAAAAACTCCAGCGCA
```

```
                                 420       430       440       450       460       470       480       490
                                 ATTTGATGCTGATGAACTTCGTGCTGCCATGAAGGGCCTTGGAACTGATGAAGATACTCTAATTGAGATTTTGGCATCAAGAA
A5089-1_CMV-F_E08.ab1(50>500) →
```

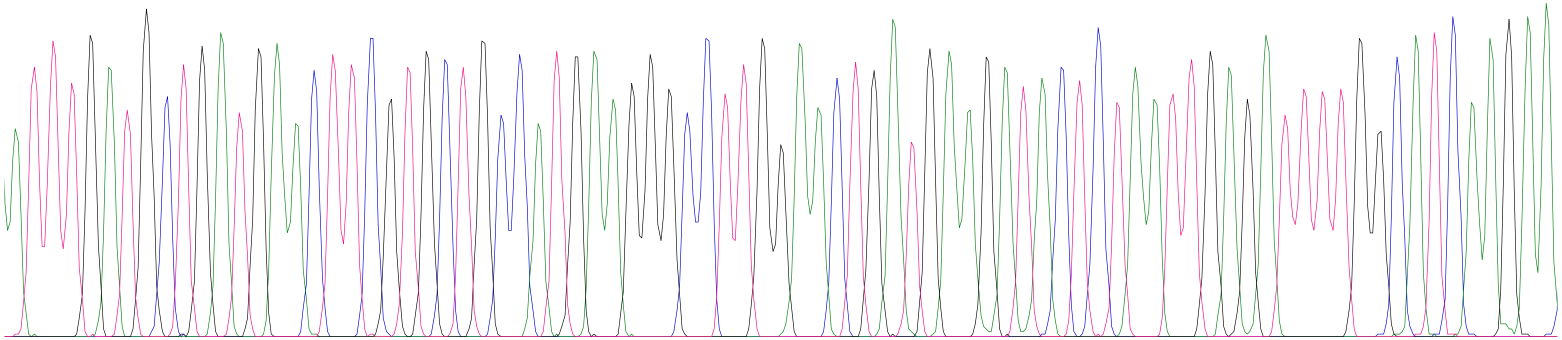

```
                                 ATTTGATGCTGATGAACTTCGTGCTGCCATGAAGGGCCTTGGAACTGATGAAGATACTCTAATTGAGATTTTGGCATCAAGAA
B5081 Homo ANXA1.seq(1>1041)  →  ATTTGATGCTGATGAACTTCGTGCTGCCATGAAGGGCCTTGGAACTGATGAAGATACTCTAATTGAGATTTTGGCATCAAGAA
```

```
                                 500       510       520       530       540       550       560       570       580
                                 CTAACAAAGAAATCAGAGACATTAACAGGGTCTACAGAGAGGAACTGAAGAGAGATCTGGCCAAAGACATAACCTCAGACACA
```

500 510 520 530 540 550 560 570 580

CTAACAAAGAAATCAGAGACATTAACAGGGTCTACAGAGAGGAACTGAAGAGAGATCTGGCCAAAGACATAACCTCAGACACA

A5089-1_CMV-F_E08.ab1(50>500) →

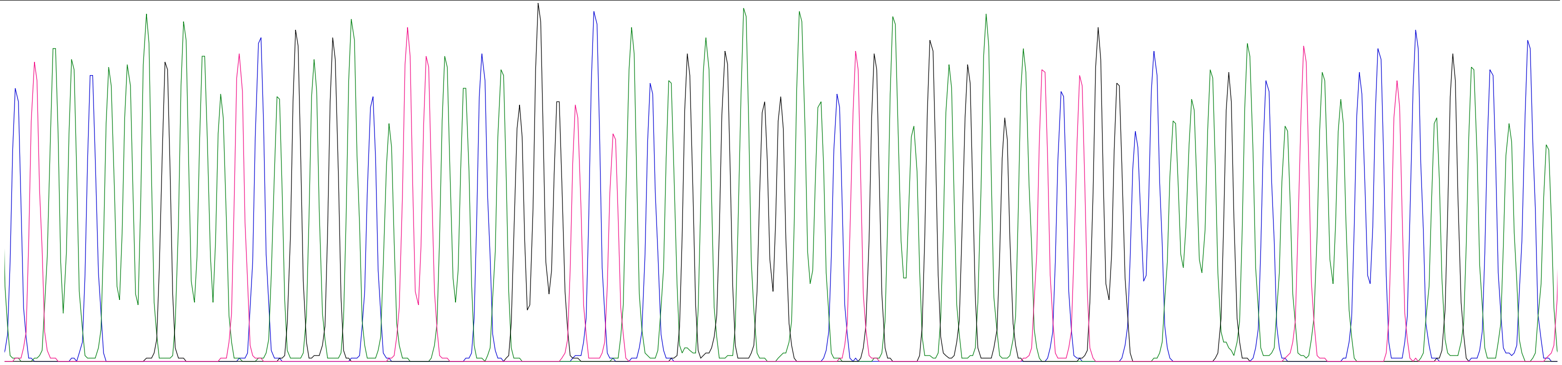

CTAACAAAGAAATCAGAGACATTAACAGGGTCTACAGAGAGGAACTGAAGAGAGATCTGGCCAAAGACATAACCTCAGACACA

B5081 Homo ANXA1.seq(1>1041) → CTAACAAAGAAATCAGAGACATTAACAGGGTCTACAGAGAGGAACTGAAGAGAGATCTGGCCAAAGACATAACCTCAGACACA

590 600 610 620 630 640 650 660

TCTGGAGATTTTCGGAACGCTTTGCTTTCTCTTGCTAAGGGTGACCGATCTGAGGACTTTGGTGTGAATGAAGACTTGGCTGA

A5089-1_CMV-F_E08.ab1(50>500) →

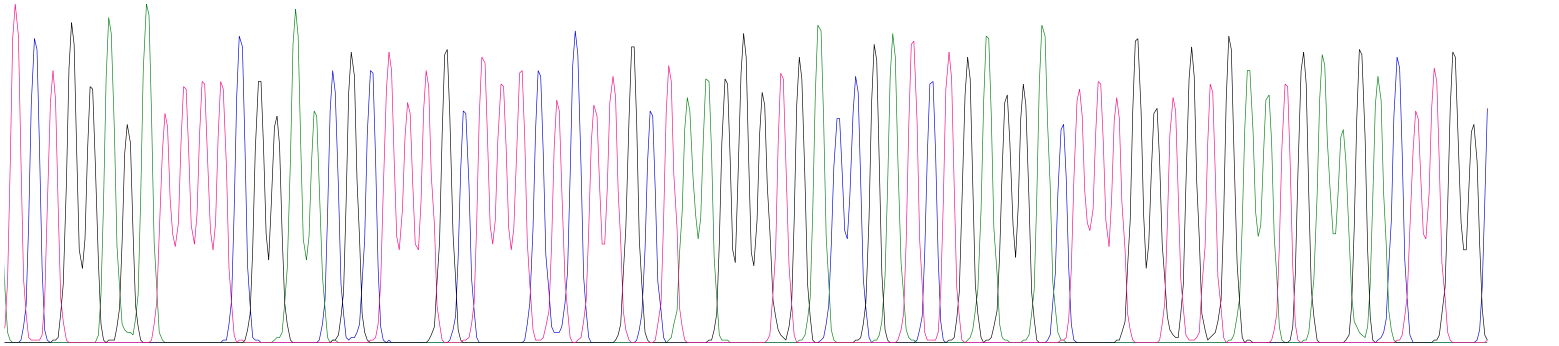

TCTGGAGATTTTCGGAACGCTTTGCTTTCTCTTGCTAAGGGTGACCGATCTGAGGACTTTGGTGTGAATGAAGACTTGG

B5081 Homo ANXA1.seq(1>1041) → TCTGGAGATTTTCGGAACGCTTTGCTTTCTCTTGCTAAGGGTGACCGATCTGAGGACTTTGGTGTGAATGAAGACTTGGCTGA

```
                       590       600       610       620       630       640       650       660
                       TCTGGAGATTTTCGGAACGCTTTGCTTTCTCTTGCTAAGGGTGACCGATCTGAGGACTTTGGTGTGAATGAAGACTTGGCTGA
A5089-1_BGH_F05.ab1(50>500)  ←
```

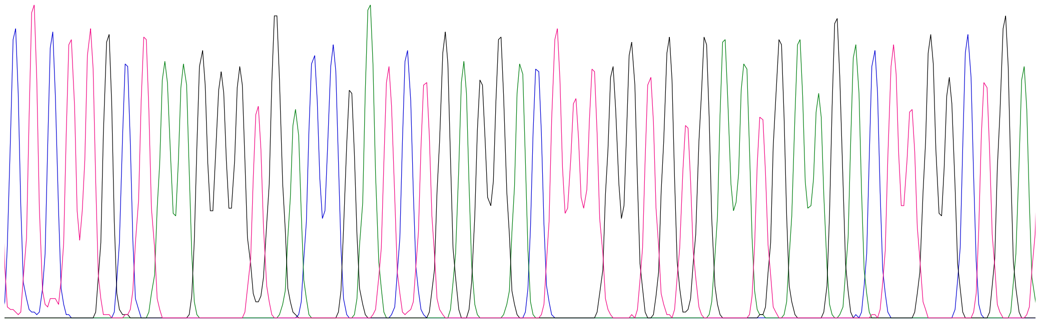

```
CTCTTGCTAAGGGTGACCGATCTGAGGACTTTGGTGTGAATGAAGACTTGGCTGA
```

```
                     670       680       690       700       710       720       730       740
                     TTCAGATGCCAGGGCCTTGTATGAAGCAGGAGAAAGGAGAAAGGGGACAGACGTAAACGTGTTCAATACCATCCTTACCACCA
B5081 Homo ANXA1.seq(1>1041)  →  TTCAGATGCCAGGGCCTTGTATGAAGCAGGAGAAAGGAGAAAGGGGACAGACGTAAACGTGTTCAATACCATCCTTACCACCA
A5089-1_BGH_F05.ab1(50>500)  ←
```

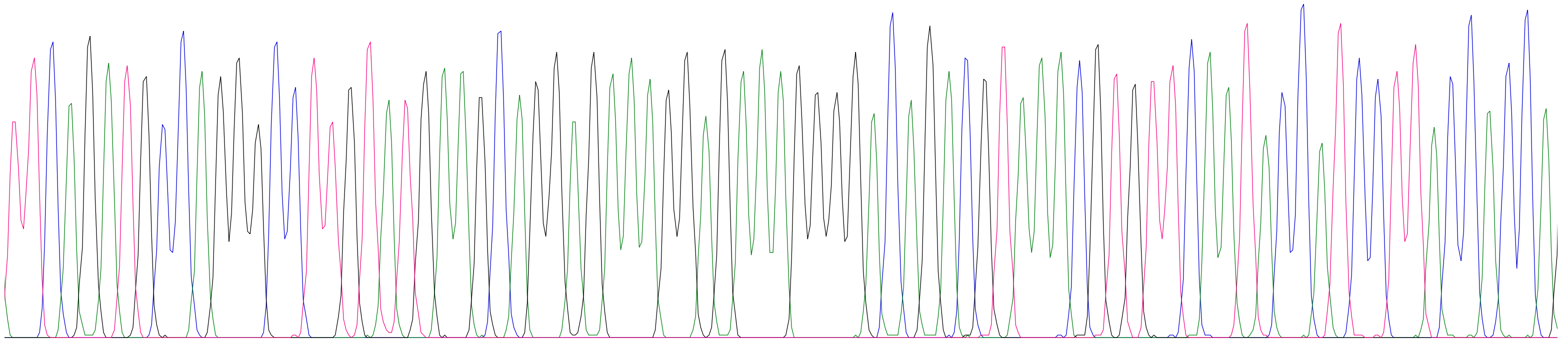

```
TTCAGATGCCAGGGCCTTGTATGAAGCAGGAGAAAGGAGAAAGGGGACAGACGTAAACGTGTTCAATACCATCCTTACCACCA
```

750 760 770 780 790 800 810 820 830

GAAGCTATCCACAACTTCGCAGAGTGTTTCAGAAATACACCAAGTACAGTAAGCATGACATGAACAAAGTTCTGGACCTGGAG

B5081 Homo ANXA1.seq(1>1041) → GAAGCTATCCACAACTTCGCAGAGTGTTTCAGAAATACACCAAGTACAGTAAGCATGACATGAACAAAGTTCTGGACCTGGAG
A5089-1_BGH_F05.ab1(50>500) ←

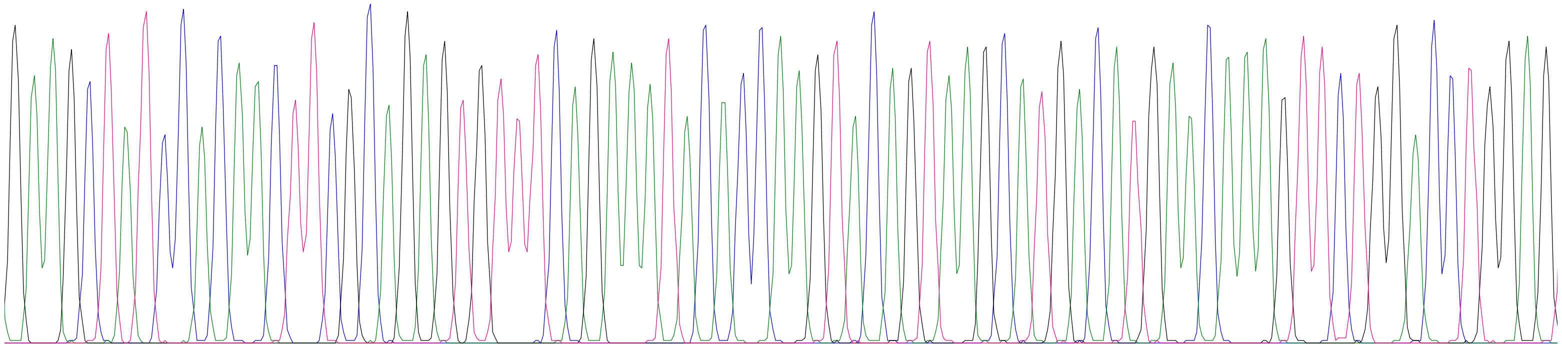

GAAGCTATCCACAACTTCGCAGAGTGTTTCAGAAATACACCAAGTACAGTAAGCATGACATGAACAAAGTTCTGGACCTGGAG

840 850 860 870 880 890 900 910

TTGAAAGGTGACATTGAGAAATGCCTCACAGCTATCGTGAAGTGCGCCACAAGCAAACCAGCTTTCTTTGCAGAGAAGCTTCA

B5081 Homo ANXA1.seq(1>1041) → TTGAAAGGTGACATTGAGAAATGCCTCACAGCTATCGTGAAGTGCGCCACAAGCAAACCAGCTTTCTTTGCAGAGAAGCTTCA
A5089-1_BGH_F05.ab1(50>500) ←

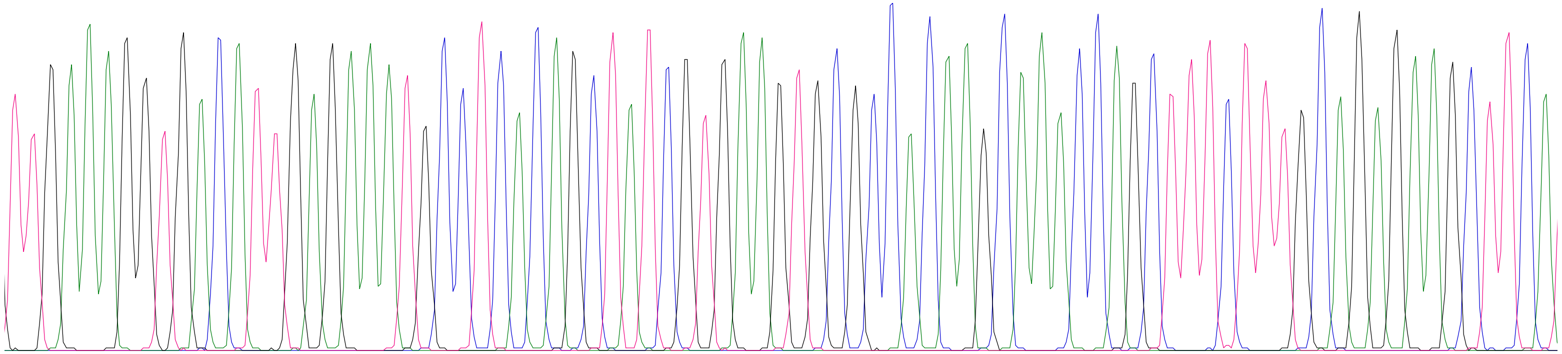

TTGAAAGGTGACATTGAGAAATGCCTCACAGCTATCGTGAAGTGCGCCACAAGCAAACCAGCTTTCTTTGCAGAGAAGCTTCA

920 930 940 950 960 970 980 990

TCAAGCCATGAAAGGTGTTGGAACTCGCCATAAGGCATTGATCAGGATTATGGTTTCCCGTTCTGAAATTGACATGAATGATA

B5081 Homo ANXA1.seq(1>1041) → TCAAGCCATGAAAGGTGTTGGAACTCGCCATAAGGCATTGATCAGGATTATGGTTTCCCGTTCTGAAATTGACATGAATGATA
A5089-1_BGH_F05.ab1(50>500) ←

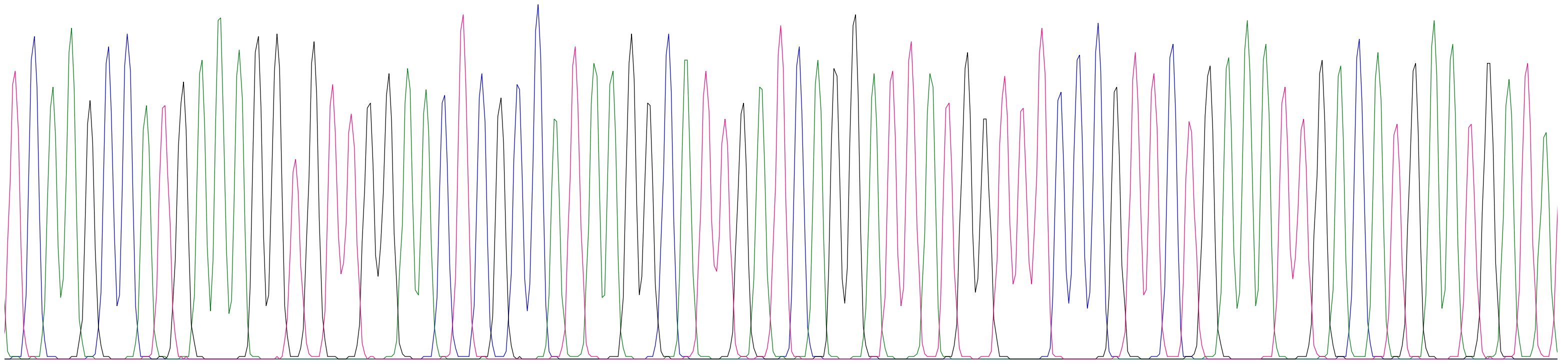

TCAAGCCATGAAAGGTGTTGGAACTCGCCATAAGGCATTGATCAGGATTATGGTTTCCCGTTCTGAAATTGACATGAATGATA

1000 1010 1020 1030 1040 1050 1060 1070

TCAAAGCATTCTATCAGAAGATGTATGGTATCTCCCTTTGCCAAGCCATCCTGGATGAAACCAAAGGAGATTATGAGAAAATC

B5081 Homo ANXA1.seq(1>1041) → TCAAAGCATTCTATCAGAAGATGTATGGTATCTCCCTTTGCCAAGCCATCCTGGATGAAACCAAAGGAGATTATGAGAAAATC
A5089-1_BGH_F05.ab1(50>500) ←

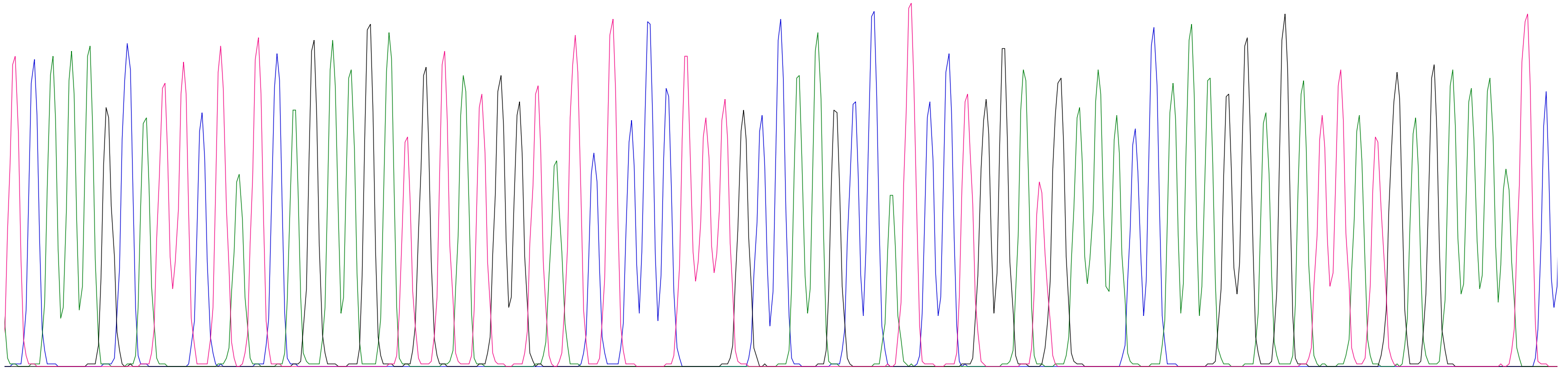

TCAAAGCATTCTATCAGAAGATGTATGGTATCTCCCTTTGCCAAGCCATCCTGGATGAAACCAAAGGAGATTATGAGAAAATC

```
                                          1080      1090      1100      1110      1120      1130      1140      1150
                                          CTGGTGGCTCTTTGTGGAGGAAACTAAGAATTCTGCAGATATCCAGCACAGTGGCGGCCGCTCGAGTCTAGAGTCCC
B5081 Homo ANXA1.seq(1>1041)          →  CTGGTGGCTCTTTGTGGAGGAAACTAA
A5089-1_BGH_F05.ab1(50>500)           ←
```

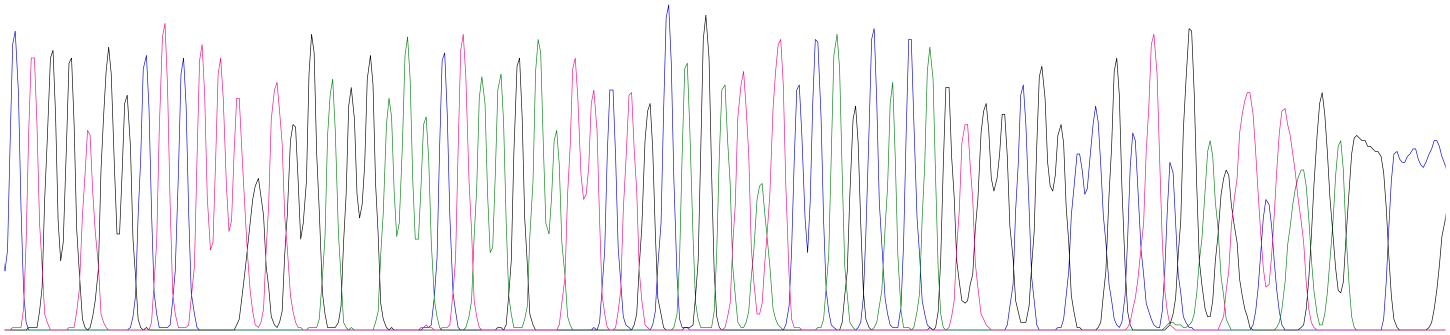

```
                                          CTGGTGGCTCTTTGTGGAGGAAACTAAGAATTCTGCAGATATCCAGCACAGTGGCGGCCGCTCGAGTCTAGAGTCCC
```
