# Supplementary material for: DCST1-AS1 Promotes TGF-β-Induced Epithelial–Mesenchymal Transition and Enhances Chemoresistance in Triple-Negative Breast Cancer Cells via ANXA1
Source: Front Oncol. 2020 Mar 12;10:280. doi: 10.3389/fonc.2020.00280 (PMC7080863; doi:10.3389/fonc.2020.00280)
Supplement: Supplementary file 5 [file Data_Sheet_4.PDF]

Supplementary material 3:

Preparation of *DCST1-AS1* Stably Transfected Cell Lines

The lentiviral fragment and negative control of *DCST1-AS1* were synthesized and packaged by Genechem (Shanghai, China). Using the GV493 plasmid as a vector, the sequence of the interference fragment was: 5'-ATCCACTGTGACTCACAAATT-3', and the negative control sequence was: 5'-TTCTCCGAACGTGTCACGT-3'. *DCST1-AS1* lentiviral overexpression vector and negative control were synthesized and packaged by GenePharma (Shanghai, China). The LV5 plasmid was used as the vector, and the empty vector was used as the negative control. MOI = 15 for BT-549 cells and 10 for MDA-MB-231 cells. 48 hours after infection, puromycin was added, and the screening concentration was 5 μg/mL, and continuous culture was performed to obtain lentivirus stable transfected cell lines.

The sequencing results of the recombinant vectors are as follows:

Interference vector sequencing result：

>PSC62906-1-H1-
F_F02.ab1NNNNNNTNAACTAAAGGATTACAAAAACAAATTACAAAAATTCAAAATTTTCGGGTTTATTACAGGGACA
GCAGAGATCCAGTTTGGTTAATTAATCGAGCGGCCGCCCCCTTCACCGAGGGCCTATTTCCCATGATTCCTTCATATTT
GCATATACGATACAAGGCTGTTAGAGAGATAATTGGAATTAATTTGACTGTAAACACAAAGATATTAGTACAAAATACG
TGACGTAGAAAGTAATAATTTCTTGGGTAGTTTGCAGTTTTAAAATTATGTTTTAAAATGGACTATCATATGCTTACCG
TAACTTGAAAGTATTTCGATTTCTTGGCTTTATATATCTTGTGGAAAGGACGAAACACCGGATCCACTGTGACTCACAA
ATTCTCGAGAATTTGTGAGTCACAGTGGATTTTTTGAATTCTCGACCTCGAGACAAATGGCAGTATTCATCCACGGATC
CTAACCCGTGTCGGCTCCAACATAACTTACGGTAAATGGCCCGCCTGGCTGACCGCCCAACGACCCCCGCCCATTGACG
TCAATAGTAACGCCAATAGGGACTTTCCATTGACGTCAATGGGTGGAGTATTTACGGTAAACTGCCCACTTGGCAGTAC
ATCAAGTGTATCATATGCCAAGTACGCCCCCTATTGACGTCAATGACGGTAAATGGCCCGCCTGGCATTGTGCCCAGTA
CATGACCTTATGGGACTTTCCTACTTGGCAGTACATCTACGTATTAGTCATCGCTATTACCATGGTCGAGGTGAGCCCC
ACGTTCTGCTTCACTCTCCCCATCTCCCCCCCCTCCCCACCCCCAATTTTGTATTTATTTATTTTTTAATTATTTTGTG
CAGCGATGGGGGCGGGGGGGGGGGGGGGGCGCGCGCCAAGGCGGGGCGGGGCGGGGCGAGGGGCGGGGCGGGGCCG
AGCCGANNGTGCGGCGNAGCCAATCAGAGCGGCGCGCTCCAAAGTTTCTTTTATGCGAGGCGCGCNCCGGCGNCCTATA
AAANCGAGCGCGNNGGCGGGCGGGAGTCGCTGCNNGCTGCTTCGCCCNTGCCCGCTCGCGCCGCCTCGAGCNNNNNNNN
CTNAANGACGGTANNCNAAGTGAACGGGNGGNNNNNTTTCTCCTCNNNGANTAGNT

PSC62906-1 ccggATCCACTGTGACTCACAAATTctcgagAATTTGTGAGTCACAGTGGATtttttg

Overexpression vector sequencing result:

10 20 30 40 50 60 70

TAACTTCGTATAGCATACATTATACGAAGTTATATTAAGGGTTCCAAGCTTAAGCGGCCGCAAAGCCCGGGAGCGCGC

A3883-2_EF1A.ab1(50>600) →

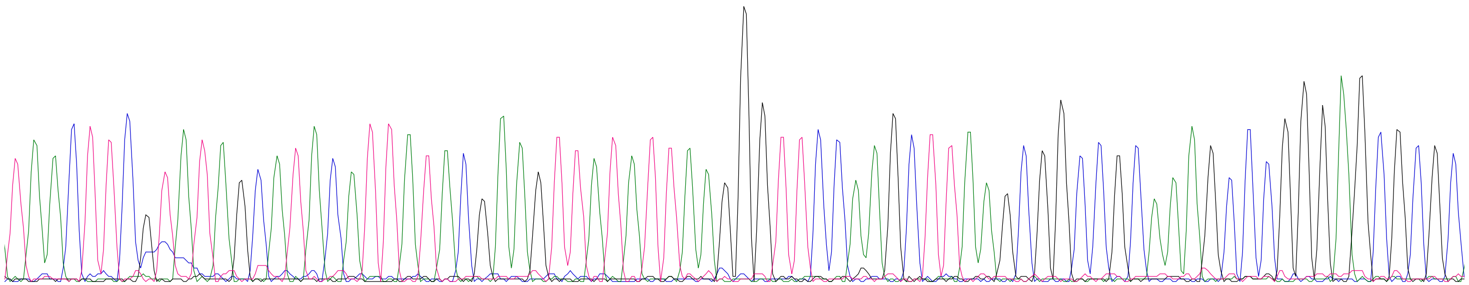

TAACTTCGTATAGCATACATTATACGAAGTTATATTAAGGGTTCCAAGCTTAAGCGGCCGCAAAGCCCGGGAGCGCGC

B4527 DCST1-AS1 Homo.seq(1>1202) → AAAGCCCGGGAGCGCGC

80 90 100 110 120 130 140 150

AGACTTGGCTGTGCGCTCGCCCGGTTCGGCCCGGCTACCTGGGGTTACTGGAGTTCCAGTAGACTACGTGGCGGAGGC

A3883-2_EF1A.ab1(50>600) →

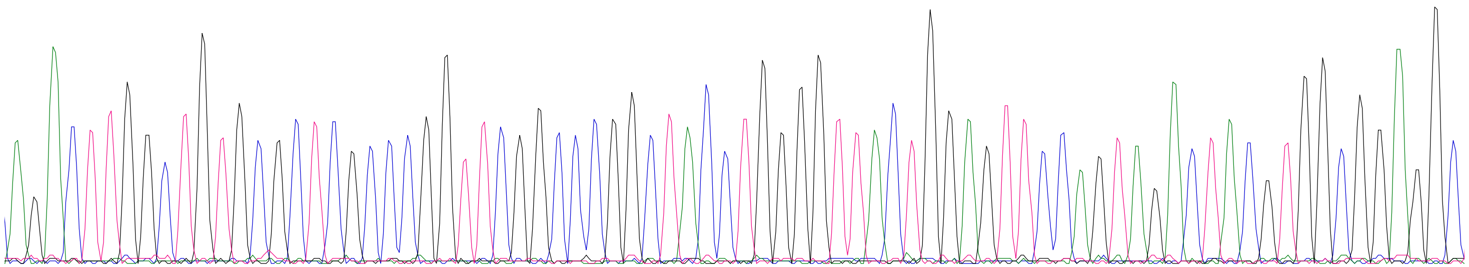

AGACTTGGCTGTGCGCTCGCCCGGTTCGGCCCGGCTACCTGGGGTTACTGGAGTTCCAGTAGACTACGTGGCGGAGGC

B4527 DCST1-AS1 Homo.seq(1>1202) → AGACTTGGCTGTGCGCTCGCCCGGTTCGGCCCGGCTACCTGGGGTTACTGGAGTTCCAGTAGACTACGTGGCGGAGGC

160 170 180 190 200 210 220 230

TGGAGCCCCCGCGCAGAGGGGAGCCGAGGAACGCGGCCCAGAGGACAGTCCGCAGCAGGGGCAGCAGCCGCATCGCCC

160 170 180 190 200 210 220 230

TGGAGCCCCCGCGCAGAGGGGAGCCGAGGAACGCGGCCCAGAGGACAGTCCGCAGCAGGGGCAGCAGCCGCATCGCCC

A3883-2_EF1A.ab1(50>600) →

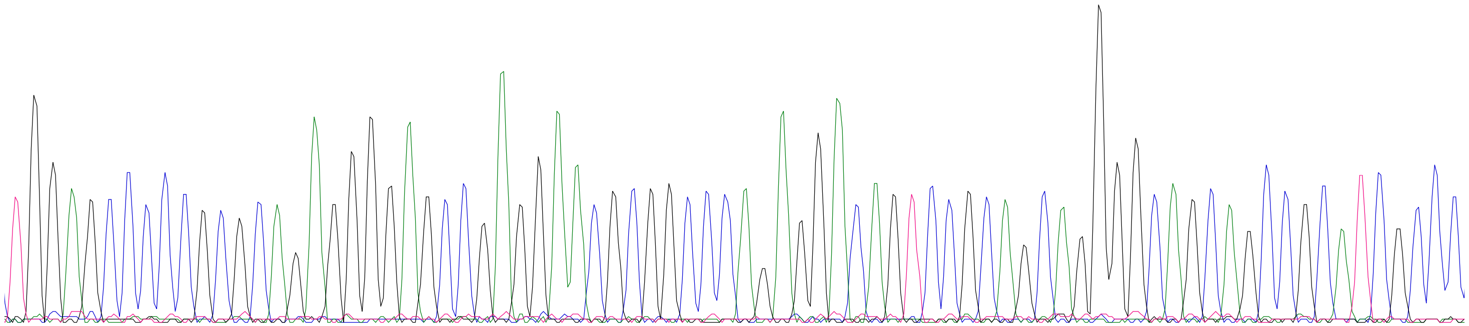

TGGAGCCCCCGCGCAGAGGGGAGCCGAGGAACGCGGCCCAGAGGACAGTCCGCAGCAGGGGCAGCAGCCGCATCGCCC

B4527 DCST1-AS1 Homo.seq(1>1202) → TGGAGCCCCCGCGCAGAGGGGAGCCGAGGAACGCGGCCCAGAGGACAGTCCGCAGCAGGGGCAGCAGCCGCATCGCCC

240 250 260 270 280 290 300 310

CCGAGGTCCGGTTTGGTCTGGCCTGGCAGGTCCCGGCCCGCTTCACAGTCGAGGAGAGAAAGGTGAGAAAACTGATGC

A3883-2_EF1A.ab1(50>600) →

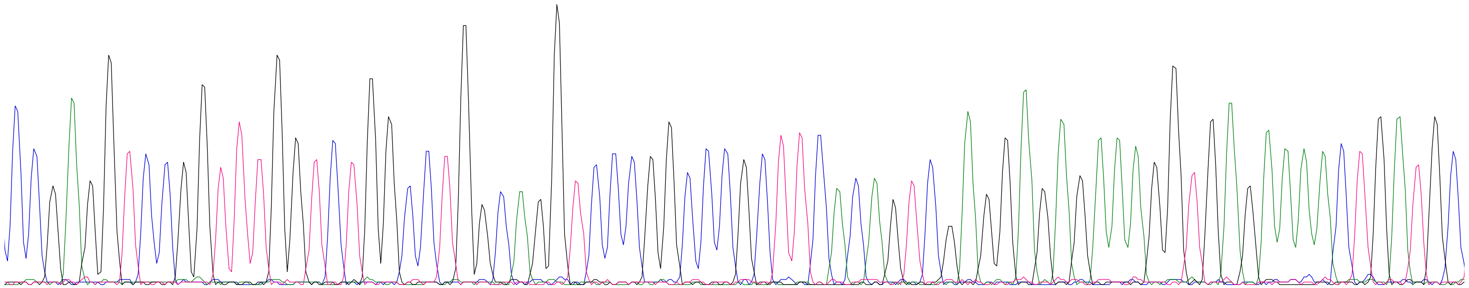

CCGAGGTCCGGTTTGGTCTGGCCTGGCAGGTCCCGGCCCGCTTCACAGTCGAGGAGAGAAAGGTGAGAAAACTGATGC

B4527 DCST1-AS1 Homo.seq(1>1202) → CCGAGGTCCGGTTTGGTCTGGCCTGGCAGGTCCCGGCCCGCTTCACAGTCGAGGAGAGAAAGGTGAGAAAACTGATGC

320 330 340 350 360 370 380 390

TTGGAGATGTGATCACTGCCCAGGGTCACCCAATGATAACATGCATGCATATGGAACTTGCTGCATGCCAGCACCATC

```
                                    320       330       340       350       360       370       380       390
                                  TTGGAGATGTGATCACTGCCCAGGGTCACCCAATGATAACATGCATGCATATGGAACTTGCTGCATGCCAGCACCATC
A3883-2_EF1A.ab1(50>600)      →
```

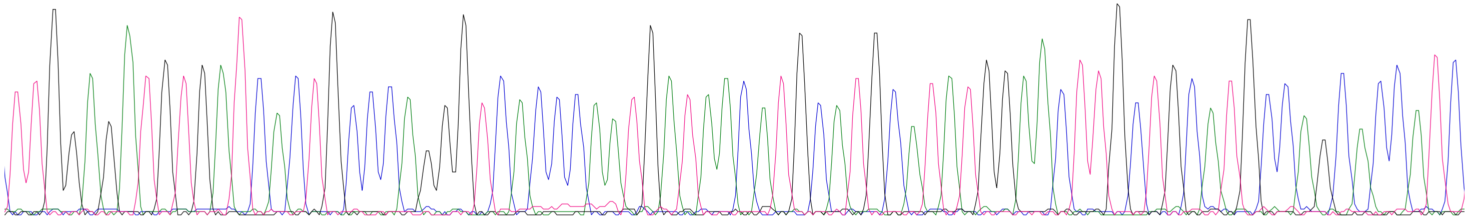

```
                                  TTGGAGATGTGATCACTGCCCAGGGTCACCCAATGATAACATGCATGCATATGGAACTTGCTGCATGCCAGCACCATC
B4527 DCST1-AS1 Homo.seq(1>1202) → TTGGAGATGTGATCACTGCCCAGGGTCACCCAATGATAACATGCATGCATATGGAACTTGCTGCATGCCAGCACCATC

                                        400       410       420       430       440       450       460
                                  TTTGCGGAGTGGCAGCAGAGTCCGTTTGCCCTGGAAAACAAATGTCCACACAGTTAGGAAGCCCAAGGGCCCTCTGCC
A3883-2_EF1A.ab1(50>600)      →
```

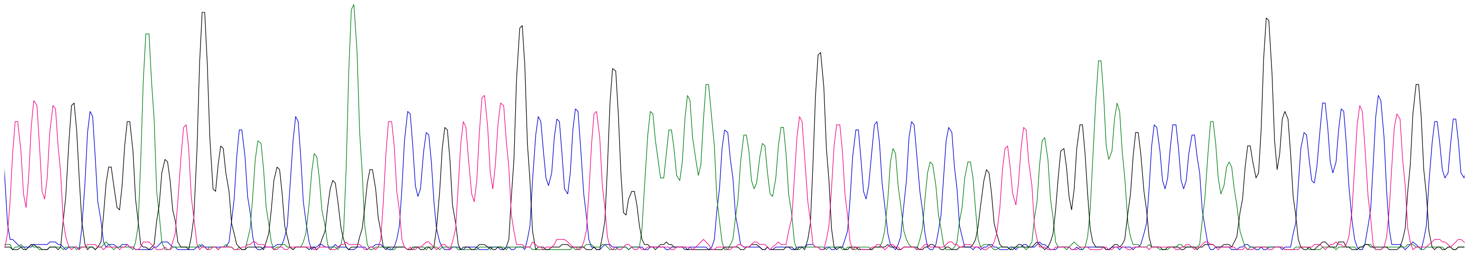

```
                                  TTTGCGGAGTGGCAGCAGAGTCCGTTTGCCCTGGAAAACAAATGTCCACACAGTTAGGAAGCCCAAGGGCCCTCTGCC
B4527 DCST1-AS1 Homo.seq(1>1202) → TTTGCGGAGTGGCAGCAGAGTCCGTTTGCCCTGGAAAACAAATGTCCACACAGTTAGGAAGCCCAAGGGCCCTCTGCC

                                  470       480       490       500       510       520       530       540
                                  CTTTCCTCTCTGCCTTCCTGGAGCATGAACCCACACAGGGCACACAGCAGCAAGGCATCCCCGGGCAGTGCCGTGCCC
```

```
                                   470       480       490       500       510       520       530       540
                                   CTTTCCTCTCTGCCTTCCTGGAGCATGAACCCACACAGGGCACACAGCAGCAAGGCATCCCCGGGCAGTGCCGTGCCC
A3883-2_EF1A.ab1(50>600)      →
```

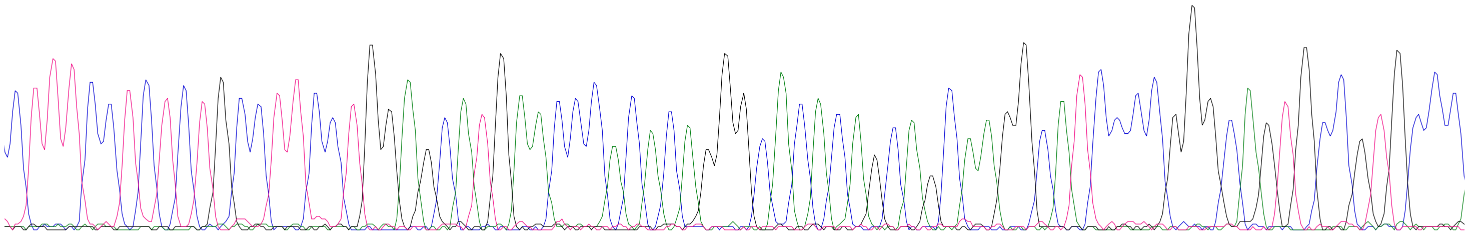

```
                                   CTTTCCTCTCTGCCTTCCTGGAGCATGAACCCACACAGGGCACACAGCAGCAAGGCATCCCCGGGCAGTGCCGTGCCC
B4527 DCST1-AS1 Homo.seq(1>1202) → CTTTCCTCTCTGCCTTCCTGGAGCATGAACCCACACAGGGCACACAGCAGCAAGGCATCCCCGGGCAGTGCCGTGCCC

                                     550       560       570       580       590       600       610       620
                                   ACTCACCAGCTTCTTCCTGCGGTCATCGATCTGGCAGAAGTTCTCCTCATCTATCCCCAAACATGGGCTTCCTTGAGG
A3883-2_EF1A.ab1(50>600)      →
```

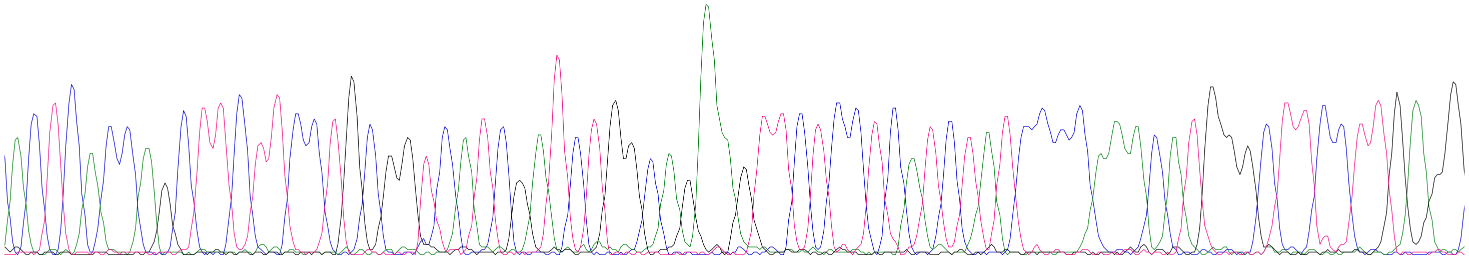

```
                                   ACTCACCAGCTTCTTCCTGCGGTCATCGATCTGGCAGAAGTTCTCCTCATCTATCCCCAAACATGGGCTTCCTTGAGG
B4527 DCST1-AS1 Homo.seq(1>1202) → ACTCACCAGCTTCTTCCTGCGGTCATCGATCTGGCAGAAGTTCTCCTCATCTATCCCCAAACATGGGCTTCCTTGAGG

                                       630       640       650       660       670       680       690       700
                                   CACAGTCATTCAACCAACCAGCCAGCATTCATTGAGCACCATCTATGTCCTGGGCACTGCTAGGGGATGGTGATAACA
```

630 640 650 660 670 680 690 700

CACAGTCATTCAACCAACCAGCCAGCATTCATTGAGCACCATCTATGTCCTGGGCACTGCTAGGGGATGGTGATAACA

A3883-2_EF1A.ab1(50>600) →

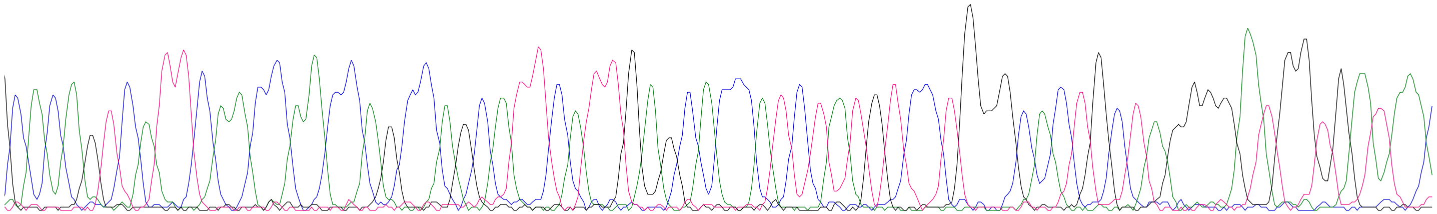

CACAGTCATTCAACCAACCAGCCAGCATTCATTGAGCACCATCTATGTCCTGGGCACTGCTAGGGGATGGTGATAA

B4527 DCST1-AS1 Homo.seq(1>1202) → CACAGTCATTCAACCAACCAGCCAGCATTCATTGAGCACCATCTATGTCCTGGGCACTGCTAGGGGATGGTGATAACA

A3883-2_PIRES2-EGFP-R.ab1(50>600) ←

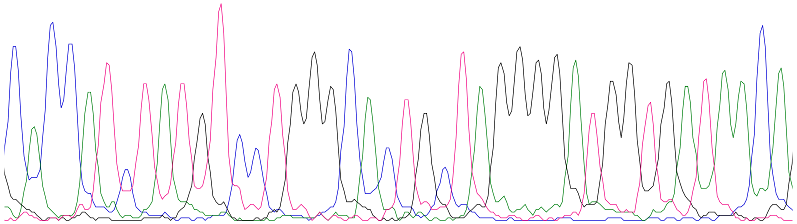

CACCATCTATGTCCTGGGCACTGCTAGGGGATGGTGATAACA

```
                                               710       720       730       740       750       760       770       780
                                               GGGAGAAGACTCTGTCCCTGCCTTCCAATTGTGTAGAGGAAGACATCCCCCTACATGATGGGTGAGACATAGCAGAAG
B4527 DCST1-AS1 Homo.seq(1>1202)          →    GGGAGAAGACTCTGTCCCTGCCTTCCAATTGTGTAGAGGAAGACATCCCCCTACATGATGGGTGAGACATAGCAGAAG
A3883-2_PIRES2-EGFP-R.ab1(50>600)        ←
```

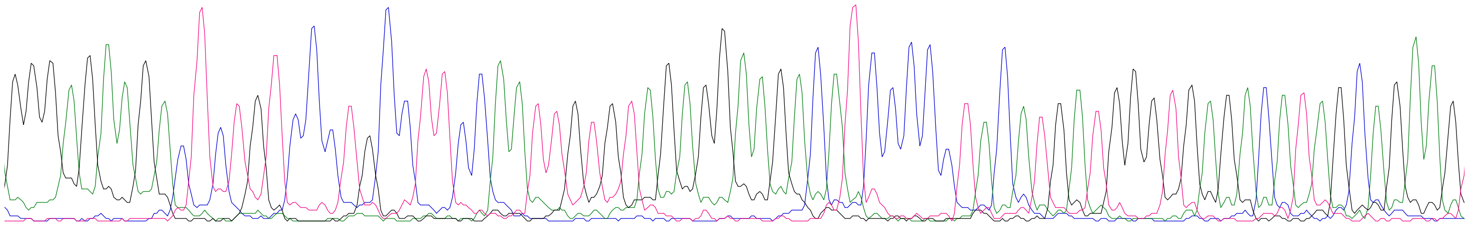

```
                                               GGGAGAAGACTCTGTCCCTGCCTTCCAATTGTGTAGAGGAAGACATCCCCCTACATGATGGGTGAGACATAGCAGAAG
```

```
                                                 790       800       810       820       830       840       850
                                               TGAGTAGGGGATGAGGTGGGGGCTCAGAGGAGGGCATGGTCAGCCTGTCTGGGAGGGAGTTGCATGTGTGCATCTGAG
B4527 DCST1-AS1 Homo.seq(1>1202)          →    TGAGTAGGGGATGAGGTGGGGGCTCAGAGGAGGGCATGGTCAGCCTGTCTGGGAGGGAGTTGCATGTGTGCATCTGAG
A3883-2_PIRES2-EGFP-R.ab1(50>600)        ←
```

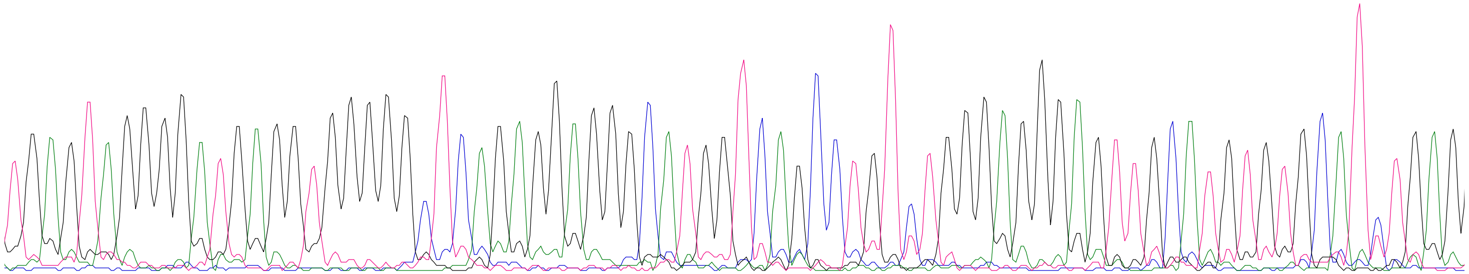

```
                                               TGAGTAGGGGATGAGGTGGGGGCTCAGAGGAGGGCATGGTCAGCCTGTCTGGGAGGGAGTTGCATGTGTGCATCTGAG
```

```
                                           860       870       880       890       900       910       920       930
                                           GTAGGGACAGGCATGCATCTTACAGGATGAATATCGAGCAGAGTTACAGAGAGGGGGAAACTCCTTGAGGTTTCAGGA
B4527 DCST1-AS1 Homo.seq(1>1202)       →   GTAGGGACAGGCATGCATCTTACAGGATGAATATCGAGCAGAGTTACAGAGAGGGGGAAACTCCTTGAGGTTTCAGGA
A3883-2_PIRES2-EGFP-R.ab1(50>600)     ←
```

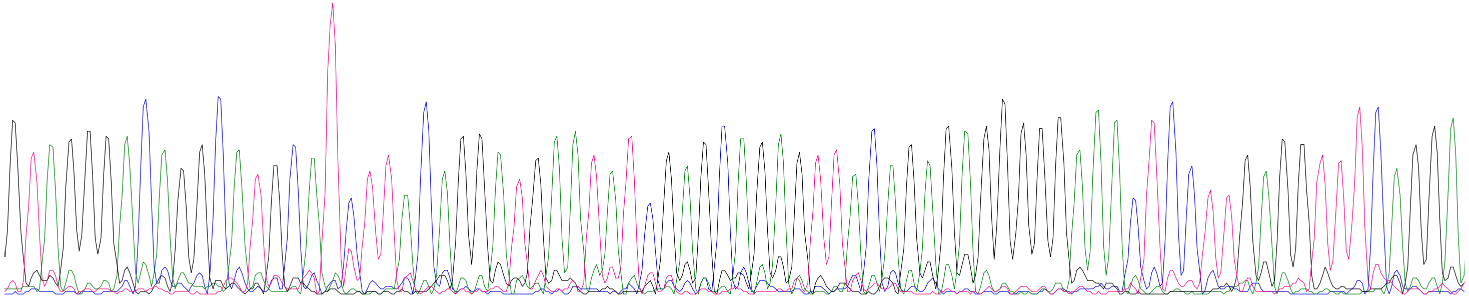

```
                                           GTAGGGACAGGCATGCATCTTACAGGATGAATATCGAGCAGAGTTACAGAGAGGGGGAAACTCCTTGAGGTTTCAGGA
```

```
                                             940       950       960       970       980       990       1000      1010
                                           ATCACCTAATCCACTGTGACTCACAAATTCCTGCCTCTTGGCTTTGCCTGCAGCATATCTCCTGGAAGTGTGCTGGGG
B4527 DCST1-AS1 Homo.seq(1>1202)       →   ATCACCTAATCCACTGTGACTCACAAATTCCTGCCTCTTGGCTTTGCCTGCAGCATATCTCCTGGAAGTGTGCTGGGG
A3883-2_PIRES2-EGFP-R.ab1(50>600)     ←
```

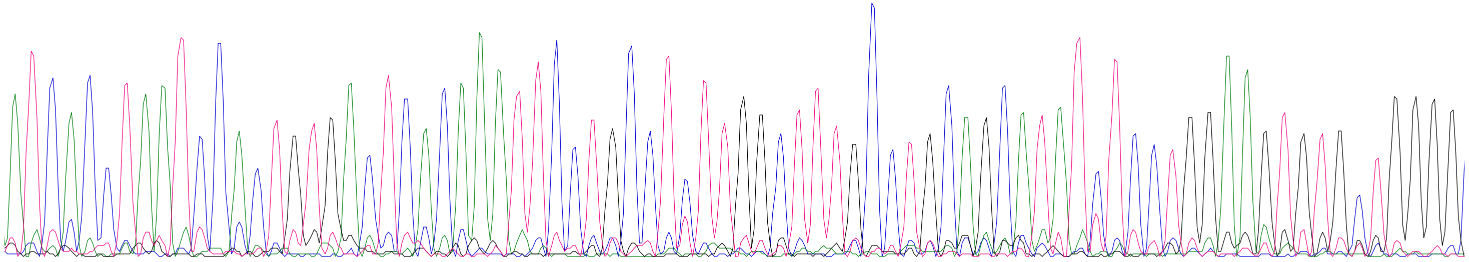

```
                                           ATCACCTAATCCACTGTGACTCACAAATTCCTGCCTCTTGGCTTTGCCTGCAGCATATCTCCTGGAAGTGTGCTGGGG
```

```
         1020      1030      1040      1050      1060      1070      1080      1090
CAAAACTCATCCCAGACCACCATCTCCATCCTCCCCCAATACACCCTGGCTCTCCCTGGCTACCCTTGAGCACGGTGC
B4527 DCST1-AS1 Homo.seq(1>1202)      →  CAAAACTCATCCCAGACCACCATCTCCATCCTCCCCCAATACACCCTGGCTCTCCCTGGCTACCCTTGAGCACGGTGC
A3883-2_PIRES2-EGFP-R.ab1(50>600)     ←
```

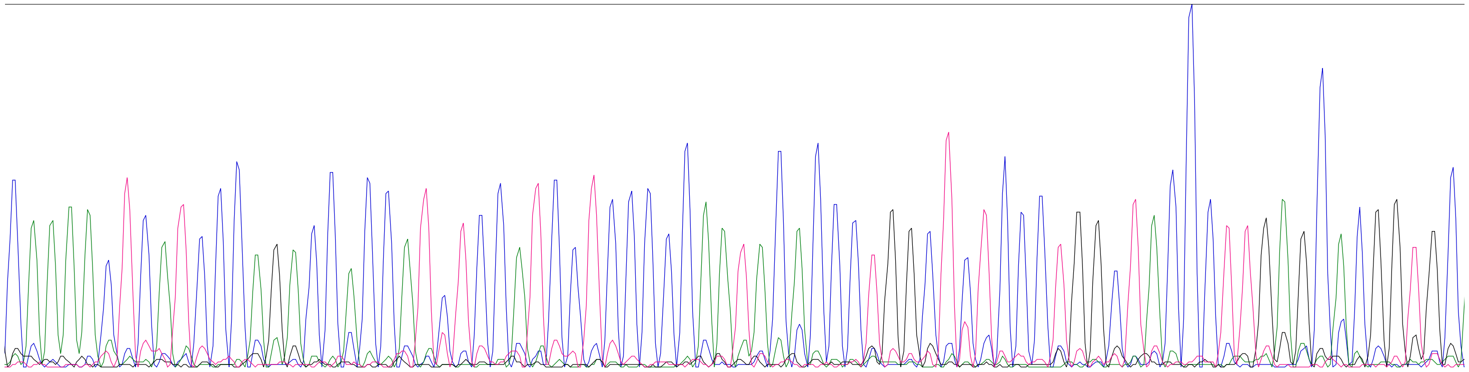

```
CAAAACTCATCCCAGACCACCATCTCCATCCTCCCCCAATACACCCTGGCTCTCCCTGGCTACCCTTGAGCACGGTGC
```

```
           1100      1110      1120      1130      1140      1150      1160      1170
ACGTGTGCATGGGTGCATGCCTGCATATATAGCTATCCCCCATGTATTTCCCAAAGCCCTACATAATGCTTCAGTTTG
B4527 DCST1-AS1 Homo.seq(1>1202)      →  ACGTGTGCATGGGTGCATGCCTGCATATATAGCTATCCCCCATGTATTTCCCAAAGCCCTACATAATGCTTCAGTTTG
A3883-2_PIRES2-EGFP-R.ab1(50>600)     ←
```

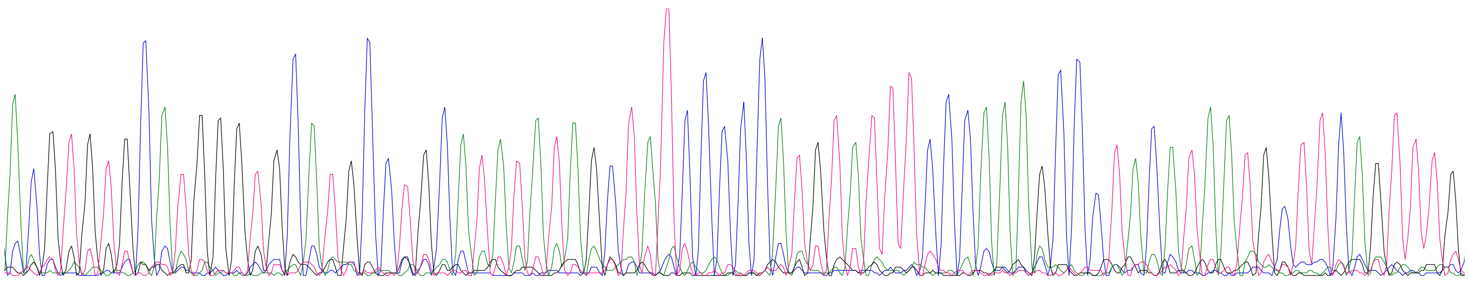

```
ACGTGTGCATGGGTGCATGCCTGCATATATAGCTATCCCCCATGTATTTCCCAAAGCCCTACATAATGCTTCAGTTTG
```

```
                                                                  1180      1190      1200      1210      1220      1230      1240
                                                      CTAAGGAAAAAATGTTAATTACTGCAAATGTGTTTAAAACTGTAAAAGTACATTAAACAAACTCTGTAAAGTGTGAAA
B4527 DCST1-AS1 Homo.seq(1>1202)               →      CTAAGGAAAAAATGTTAATTACTGCAAATGTGTTTAAAACTGTAAAAGTACATTAAACAAACTCTGTAAAGTGTGAAA
A3883-2_PIRES2-EGFP-R.ab1(50>600)              ←
```

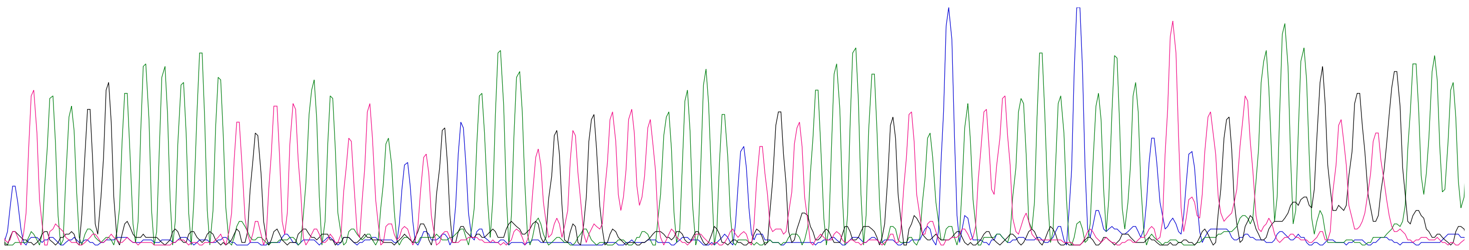

```
                                                      CTAAGGAAAAAATGTTAATTACTGCAAATGTGTTTAAAACTGTAAAAGTACATTAAACAAACTCTGTAAAGTGTGAAA
```

```
                                                      1250      1260      1270      1280      1290      1300
                                                      AAAAAAAAAAAAAAAGGATCCGACACTCTCTACTGATACTCTCACTAACGTTC
B4527 DCST1-AS1 Homo.seq(1>1202)               →      AAAAAAAAAAAAAAA
A3883-2_PIRES2-EGFP-R.ab1(50>600)              ←
```

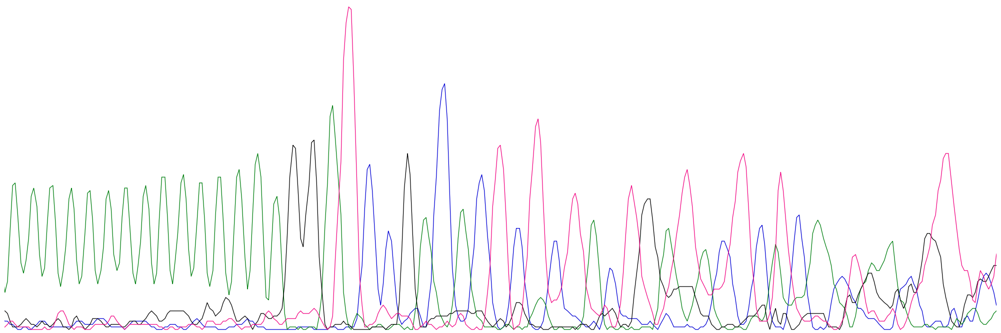

```
                                                      AAAAAAAAAAAAAAAGGATCCGACACTCTCTACTGATACTCTCACTAACGTTC
```
